# Clinically relevant mutations in regulatory regions of metabolic genes facilitate early adaptation to ciprofloxacin in *Escherichia coli*

**DOI:** 10.1093/nar/gkae719

**Published:** 2024-08-24

**Authors:** Arijit Pal, Dipannita Ghosh, Pratyusha Thakur, Priya Nagpal, Madhumathi Irulappan, Karthik Maruthan, Sanket Mukherjee, Nikita G Patil, Tanmay Dutta, Balaji Veeraraghavan, Perumal Vivekanandan

**Affiliations:** Kusuma School of Biological Sciences, Indian Institute of Technology Delhi, Hauz Khas, New Delhi 110016, India; Department of Zoology, Raiganj Surendranath Mahavidyalaya, Sudarshanpur, Raiganj, Uttar Dinajpur, West Bengal733134, India; Kusuma School of Biological Sciences, Indian Institute of Technology Delhi, Hauz Khas, New Delhi 110016, India; RNA Biology Laboratory, Department of Chemistry, Indian Institute of Technology Delhi, Hauz Khas, New Delhi 110016, India; Kusuma School of Biological Sciences, Indian Institute of Technology Delhi, Hauz Khas, New Delhi 110016, India; Department of Clinical Microbiology, Christian Medical College, Vellore, Tamil Nadu, India; Department of Clinical Microbiology, Christian Medical College, Vellore, Tamil Nadu, India; Kusuma School of Biological Sciences, Indian Institute of Technology Delhi, Hauz Khas, New Delhi 110016, India; Kusuma School of Biological Sciences, Indian Institute of Technology Delhi, Hauz Khas, New Delhi 110016, India; Amity Institute of Virology and Immunology, Amity University, Noida, Uttar Pradesh, India; RNA Biology Laboratory, Department of Chemistry, Indian Institute of Technology Delhi, Hauz Khas, New Delhi 110016, India; Department of Clinical Microbiology, Christian Medical College, Vellore, Tamil Nadu, India; Kusuma School of Biological Sciences, Indian Institute of Technology Delhi, Hauz Khas, New Delhi 110016, India

## Abstract

The genomic landscape associated with early adaptation to ciprofloxacin is poorly understood. Although the interplay between core metabolism and antimicrobial resistance is being increasingly recognized, mutations in metabolic genes and their biological role remain elusive. Here, we exposed *Escherichia coli* to increasing gradients of ciprofloxacin with intermittent transfer-bottlenecking and identified mutations in three non-canonical targets linked to metabolism including a deletion (tRNA-ArgΔ414-bp) and point mutations in the regulatory regions of *argI* (ARG box) and *narU*. Our findings suggest that these mutations modulate arginine and carbohydrate metabolism, facilitate anaerobiosis and increased ATP production during ciprofloxacin stress. Furthermore, mutations in the regulatory regions of *argI* and *narU* were detected in over 70% of sequences from clinical *E. coli* isolates and were overrepresented among ciprofloxacin-resistant isolates. In sum, we have identified clinically relevant mutations in the regulatory regions of metabolic genes as a central theme that drives physiological changes necessary for adaptation to ciprofloxacin stress.

## Graphical Abstract

**Graphical Abstract:**
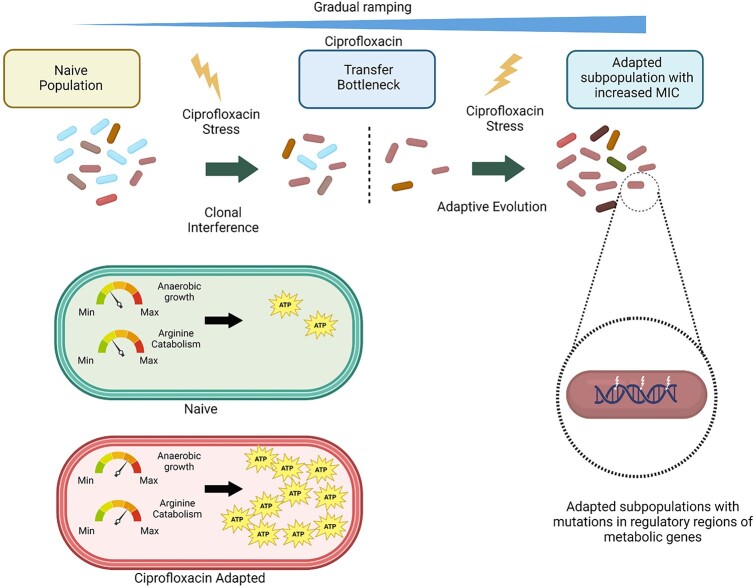

## Introduction

The rapid global dissemination of ciprofloxacin resistance in *Escherichia coli* has significantly compromised empirical treatment options, making it extremely difficult for clinicians to treat infections (1). Molecular investigations have identified several mutations in QRDR (quinolone resistance determining region) of gyrase (*gyrA* and *gyrB*), topoisomerase IV (*parC* and *parE*), and regulators of ciprofloxacin influx-efflux systems in ciprofloxacin resistance^1^. Till date, these resistance determinants have largely been reported from either clinical isolates or in laboratory-adapted strains when challenged with extremely high concentrations (i.e. 16.0× or more) of ciprofloxacin (2–4). Repeated exposures to sub-MIC antibiotics may induce a meta-stable transient resistance phenotype, enabling the bacteria to withstand supra-MIC doses (5), and stochastic bet-hedging during the antibiotic challenge may rescue the population by developing partially heritable phenotypic plasticity (6). Nonetheless, the genomic landscapes associated with the early events in adaptation that subsequently facilitate permanent resistance development remain elusive.

A ‘general stress response’ that triggers DNA-repair mechanisms and adaptivity via., supporting a hypermutated state has been linked to survival under ciprofloxacin stress (7,8). However, ciprofloxacin-induced mutations reported earlier are limited to classical targets (9). While the association between adaptation to ciprofloxacin and mutagenic processes is well-documented, mutations that occur during the early events in adaptation and their biological role remain unknown.

Whole genome transcriptomics approaches have identified a strong association between antibiotic-induced general stress response and metabolic rewiring (10,11). Despite the increasingly recognized interplay between metabolism and antibiotic stress, specific mutations in non-canonical targets and their role in adaptation remain poorly understood. Mutations in core metabolic genes are rare and often drift to extinction before being established (12). We, therefore, used transfer bottlenecking (13), a widely used method to study the evolutionary trajectory of bacteria under progressively increasing selection pressure to identify non-canonical mutations linked to metabolism and their role in adaptive evolution during ciprofloxacin challenge.

We captured the changing genomic landscapes across the different selection levels and demonstrated a strong selective sweep at 1.0× MIC ciprofloxacin that led to a yet unknown intergenic mutation upstream of *narU* (a nitrate transporter). This mutation upstream of *narU* was consistently detected at all selection levels and it was associated with up to 10-fold higher expression of the *narUZYWV* operon, resulting in a shift to anaerobic metabolism. We also identified a ‘ARG box’ mutation at the highest selection level that facilitates increased ATP production under oxygen limiting conditions through the arginine-deiminase (ADI) pathway in the adapted subpopulation. Mutations in laboratory-adapted subpopulations may not necessarily be detected in clinical isolates. Nevertheless, mutations in the regulatory regions of *narU* and *argI* were detected in at least 72% and 75% respectively of multidrug-resistant *E. coli* clinical isolates from a tertiary care unit and the sequences from the PATRIC database, suggesting these mutations are clinically relevant and are more common than previously recognized. In sum, this work sheds light on a close association between mutations in metabolic genes and early events leading to antibiotic stress response.

## Materials and methods

### Determination of antimicrobial susceptibility of *Escherichia coli* BW25113 to ciprofloxacin

Antimicrobial susceptibility of *E. coli* BW25113 (#cat OEC5042, Keio Knock-out Collection, Horizon Discovery, Cambridge, UK) to ciprofloxacin (HiMedia Laboratories Pvt. Ltd, Mumbai, India) was determined in both broth micro-dilution (as per CLSI guidelines) and LB agar plate-based method (14,15). In the agar plate-based method, approximately 10^6^ cells were grown onto LB agar supplemented with 2 to 64 ng/ml of ciprofloxacin. The minimum concentration of ciprofloxacin that can inhibit more than 99.99% growth was identified as minimum inhibitory concentration (MIC) of ciprofloxacin.

### Generation of adaptive-stress response model

*Escherichia coli* BW25113 was challenged with increasing gradients of ciprofloxacin from 0.5× MIC to 8.0× MIC following the scheme adapted by an earlier study with some modifications (16). First, *E. coli* cells were passaged four times onto ciprofloxacin-free LB agar plates, allowing the intra-population diversity to achieve an equilibrium. Then, the cells were challenged twice (cycle 1 and cycle 2: each comprising of 4 days corresponds to ∼100 generations) to each of the selection levels with a single day in between experimental bottlenecking (medium inoculum size i.e. 10^3^ CFU/ml) with selection pressures corresponding to the previous level (see Supplementary Figure S1 for experimental scheme). Medium transfer bottleneck not only promotes clonal interference but also increases the probability of transfer of high-fitness mutants to the next level, thereby improve relative frequency (13). After experimental bottlenecking the most numerically-dominant morphotype was selected for re-exposure. This selection bias not only reduced intra-population diversity but also ensured selection of the fittest lineage and filtered out the chances of capturing spontaneous mutations that may contribute to noise. The same scheme was followed to adapt *E. coli* BW25113 *narU* knockout strain (i.e. Δ*narU* strain; #cat OEC4987, Keio Knock-out Collection, Horizon Discovery, Cambridge, UK) to increasing concentration gradients of ciprofloxacin.

### Validation of generated adaptive-stress response model

To validate the generated adapted strains, survival ratio, enrichment of the population with less susceptible subtypes along the time and across the selection levels were estimated at the end of the four selection levels: 0.5×, 1.0×, 2.0× and 8.0× MIC (i.e. a sub-MIC level, MIC level and two supra-MIC levels) using previously described protocols (17) and E-strip test (18). Before transitioning to a higher selection level, pre-adaptation to the lower selection levels was evaluated by Luria–Delbruck Fluctuation assay (19). The adapted strains were passaged four times onto ciprofloxacin-free LB agar plates after each cycle of exposures and MIC was re-evaluated in LB agar plate-based method to evaluate stability of ciprofloxacin susceptibility (17). Finally, growth-related fitness cost was estimated with adapted strains, maximum growth rate (μ) and fitness co-efficient (W) were estimated using standard methods (20).

### Native DNA sequencing

Genomic DNA was extracted using the phenol:chloroform method. The gDNA was quantified using Qubit dsDNA BR Assay kit (Thermo Fischer Scientific, MA, USA) in Qubit 2.0 Fluorometer. The quality was assessed using Implen Nanophotometer N60 (Implen, Munich, Germany) and integrity of the extracted gDNA was evaluated by horizontal 0.8% agarose gel electrophoresis. The native DNA sequencing library was prepared with PCR-free native barcode expansion kit (EXP-NBD104) and ligation sequencing kit (SQK-LSK109) from Oxford Nanopore Technologies (ONT, Oxford, UK). Briefly, 1μg of gDNA from each of the adapted strains was end-prepped using NEBNext FFPE DNA repair mix (#cat E7360) and NEBNext Ultra II End repair/dA-tailing module (#cat E7595) from New England Biolabs (NEB, MA, USA). Then, each sample was ligated with unique barcodes provided by ONT. The barcoded gDNA were pooled in equimolar quantities and were subjected to adaptor ligation and clean-up. AMPure XP beads (Beckman Coulter Inc., CA, USA) were used for clean-up. Finally, the library was loaded onto an R9.4.1 flow cell (FLO-MIN106D) and sequenced on GridIONx5 (ONT, Oxford, UK). All sequencing data was submitted to Sequence Read Archive (SRA) repo What Flips the Switch? Signals and Stress Regulating Extraintestinal Pathogenic Escherichia coli Type 1 Fimbriae (Pili). sitory under BioProject, National Center for Biotechnology Information (NCBI).

### Identification of single nucleotide polymorphisms (SNPs), small insertion-deletions (INDELs) and structural variants (SVs)

The raw FAST5 files generated from native DNA sequencing were used for basecalling in Guppy version 6.1.7 using the high accuracy (HAC) model (dna_r9.4.1_450bps_hac.cfg). Then, adapter trimming, demultiplexing and concatenation was done to generate FASTQ files. The quality assessment was performed with FASTQ files in Nanoplot (21). SNPs and INDELs were called in Snippy 4.6.0 using the FASTQ files with *Q* score ≥10. The same single-end long reads in FASTQ files having *Q* score ≥10 were used for mapping to the reference genome (*E. coli* BW25113; Gene Accession: CP009273.1) in Minimap2 version 2.17-r941 (22). Then, the mapped BAM files were sorted and indexed in Samtools version 1.16 (23). Structural variants were called in Sniffles v2_2.0.7 (24) and were subjected to filtering using bcftools (25) view with parameter –i ‘AF > 0.3 && SVLEN <1 000 000 && SVLEN> -1 000 000’. The circular and linear plots with SNPs, INDELs and SVs were generated using Dna_Features_Viewer in python library (https:// github.com/Edinburg-Genome-Foundry/DnaFeaturesViewer).

The SNPs, INDELs and SVs identified were reconfirmed using Sanger sequencing. Briefly, primers, mentioned in Supplementary Table S1, were designed from the flanking regions of the mutations and the targeted regions were PCR-amplified in Agilent Sure Cycler 8800 (Agilent Technologies, CA, USA). PCR products were purified with QIAquick PCR purification kit (Qiagen, MD, USA) and sequenced with both the primers in 3100-Avant Genetic Analyzer (PE Applied Biosystems Inc., MA, USA) using BigDye Terminator v3.1 Cycle Sequencing Kit (Applied BioSystems, MA, USA). Finally, sequence reads were properly annotated and submitted to GenBank database of NCBI.

### Inversion assay

To further validate the presence of *fimS* inversion, an inversion assay described previously (26) was designed. Briefly, PCR amplification was performed with either two forward or reverse primer sets, listed in Supplementary Table S1, using DNA extracted from 2.0× survived and 8.0× survived subpopulations as template.

### qPCR assay

Quantitative PCR (qPCR) assays were performed to investigate the impact of mutations on the transcript level of efflux pump genes and their regulators (*acrB*, *acrR* and *marA*), *narUZYWV* operon genes (*narU, narZ*, *narY*, *narW* and *narV*), exonuclease VII (*xseA*), inhibitor of RNase E (*rraB*) and genes related to anaerobic metabolism (*g6pd*, *aldB* and l*dhA*), arginine utilization and metabolism (*argI*, *adiA*, *astA* and *arcA*), and biofilm formation (*fimB*, *fimA* and *entB*). The primer sets used in this study were designed through Primer3 v0.4.0 (bioinfo.ut.ee/primer3-0.4.0/) (mentioned in Supplementary Table S1). The relative expression level of transcripts was estimated in 2^−ΔΔCT^ method. Briefly, total RNA was extracted in TRIzol method (Ambion Inc., Life Technologies, CA, USA) and DNase I (New England Biolabs, MA, USA)-treated RNA samples were reverse transcribed to cDNA by iScript cDNA synthesis kit (Bio-rad Laboratories, CA, USA). qPCR was performed using TB Green Premix Ex Taq II (Takara Bio Inc., Shiga, Japan). 16s ribosomal RNA was used internal reference gene for data normalization. A no template control (NTC) and a control without RT were used for all qPCRs. All test samples were run in triplicates.

### Construction of plasmids and promoter activity assay

To link the altered expression of transcripts with the acquired mutations in the corresponding regulatory regions, the promoter activity assays were performed with both wild type and mutated promoters. Briefly, the wildtype promoter region of *narUZYWV* operon and *argI* was amplified using specific sets of primers listed in Supplementary Table S1 and after double digestion with *KpnI* and *SalI* the amplified fragments were cloned into pRU1097 vector with *gfp* as the reporter gene downstream of the multiple cloning site (gifted from Phillip Poole, Addgene plasmid# 14462). Then, to induce mutation at the requisite position at the promoter, site directed mutagenesis was performed using the specific sets of primers listed in Supplementary Table S1. Both wild type and mutated promoters (i.e. *argI* G4468182A and *narU* upstream element T1538580C) were confirmed by Sanger sequencing. Finally, after transforming *E. coli* DH5α with the constructed clones, the transformed *E. coli* strains were used for promoter activity assay following the protocol described earlier (27). Briefly, the transformed *E. coli* strains were inoculated in 2 mL LB broth and were grown overnight at 37°C with shaking at 180 rpm. The optical density was measured at λ = 600 nm in the Multiskan GO Microplate Spectrophotometer (Thermo Fisher Scientific, MA, USA). GFP fluorescence was measured using BioTek Cytation 5 Cell Imaging Multimode Reader (Agilent Technologies, CA, USA) equipped with excitation filters 485 nm (for GFPmut3.1 reporter gene), and emission filter 510 nm, respectively. The specific fluorescence was measured and normalization was done by dividing the fluorescence of by the OD.

### Assessment of microaerophilic growth

The adapted strains were cultured under microaerophilic conditions using the candle-jar method (28). Sealed, airtight candle jars provide an atmosphere with reduced oxygen tension to support the growth of anaerobes and microaerophilic organisms. Sodium bicarbonate (0.042%) is added to the medium that serves as a buffer (29). Turbidity was measured in Multiskan GO Microplate Spectrophotometer (Thermo Fisher Scientific, MA, USA) at optical density: λ = 600 nm to compare anaerobic growth with that under aerobic conditions.

### Assessment of biofilm formation

Biofilm formation was assessed with microtiter dish biofilm formation assay described earlier (30). Laser scanning confocal microscopy and transmission electron microscopy were performed as described earlier (31,32) to visualize biofilm and fimbriae, respectively.

### Estimation of ATP and total protein per cell

ATP production and total protein content were estimated using BacTiter-Glo Microbial cell viability assay kit (Promega Corporation, WI, USA) and bicinchoninic acid (BCA) protein assay kit (Pierce, Thermo Scientific, MA, USA), respectively. Briefly, 0.5 McFarland bacteria (corresponds to 1.5 × 10^8^ CFU/ml) was inoculated in 2 ml Luria broth and incubated overnight at 37°C with orbital shaking at 220 rpm. The cells were harvested by centrifugation @ 9800×g for 10 min and resuspended in 2 ml fresh Luria broth. The optical density was measured at λ = 600 nm in Multiskan GO Microplate Spectrophotometer (Thermo Fisher Scientific, MA, USA). The ATP production was estimated by measuring relative luminescence in BioTek Cytation 5 Cell Imaging Multimode Reader (Agilent Technologies, CA, USA). The cell lysates were prepared in Qsonica Q125 sonicator (Cole-Parmer India Pvt. Ltd, Mumbai, India) using the following parameters: four 30 s cycles of 40 W sound energy with 10 s gap in between the cycles. The total protein content of the cell lysates was estimated in bicinchoninic acid (BCA) method. To estimate ATP/10^3^ cells and total protein content/10^9^ cells, the relative luminescence and absorbance values obtained from the end-point assays were divided by the cell numbers corresponding to their optical densities.

### Construction of a tRNA-ArgΔ414-bp knock-out (KO) mutant *E. coli*

To understand the functional impact of 414 bp deletion in t-RNA-Arg gene on total protein content, a tRNA-ArgΔ414-bp knock-out (KO) mutant *E. coli* was constructed following the protocol described earlier (33). Briefly, gene knock-out was performed using λ Red-mediated recombination with fragments generated by PCR using gene specific primers mentioned in Supplementary Table S1. Plasmid pSIM6 express the red proteins under control of the λ phage pLpromoter, which is induced by heat-shock, was transformed into the *E. coli* BW1125 strain. Cells were inoculated in 5 ml of LB medium supplemented with ampicillin (100 μg/ml) and were grown overnight at 30°C with shaking at 180 rpm. Primary culture was diluted 1:100 into fresh LB medium and allowed to grow in the same conditions till OD_600_ became ∼0.5. After that, the culture was transferred to a 42°C-water bath shaker for 15 min. The culture flask was immediately transferred to an ice bath after the heat induction and kept it for 10 min. Cells were collected through centrifugation at 8000 rpm for 5 min at 4°C. Cells were made electro**-**competent by washing 3 times with double distilled water. Purified PCR product was fused into the competent cells using Bio-Rad micropulser (1.0 mm electroporation cuvettes were used). Transformed cells were grown in LB agar plate containing kanamycin (50 μg/ml) as a selection marker. Colonies were screened to check the absence of the gene. Further confirmation was done by Sanger sequencing. Total protein content per 10^9^ cells was also estimated.

### Estimation of enzyme activity and free l-arginine

The enzyme activity of isocitrate dehydrogenase was estimated in spectrophotometric method using a kit from Elabscience, TX, USA. The amount of free l-arginine present within the adapted strains was estimated using l-arginine-urea-ammonia assay kit (Megazyme, Wicklow, Ireland).

### Retrieval of *E. coli* WGS data and analysis of variants in clinical isolates

A total of 661 Whole Genome Sequence assemblies of *E. coli* were retrieved from the Pathosystems Resource Integration Center (PATRIC) database using the following filters—Host Group: Human, Genome Quality: Good and Genome Status: Complete (Supplementary Table S2). Specifically, to ensure high-quality data relevant to clinical settings, we removed any sequences that did not have any isolation source information. Additionally, we analysed whole-genome sequencing data of multidrug-resistant clinical isolates of *E. coli* (*n* = 89) from Christian Medical College, Vellore, India (Supplementary Table S3); 60 of these isolates were analysed previously for antibiotic resistance genes (34). For further validation, we also analyzed whole-genome sequencing data of fluoroquinolone-resistant *E. coli* (*n* = 113) from healthy children reported from China (35). We used snippy 4.6.0 to scan unique nucleotide positions having mutations and used *E. coli* K-12 MG1655 (NC_000913.3) as a reference for mapping (36). To focus on the variants relevant to our study, we filtered for intergenic regions between *argI-rraB*, and intergenic regions upstream of *narU* (*narU-yddK/J*) and t-RNA-Arg coding region. The unique nucleotide positions with mutations were identified for each of the above-mentioned regions and the entire intergenic sequences.

## Results

### Selection of ciprofloxacin-adapted high-fitness subpopulations using transfer bottlenecking

**Figure 1.**
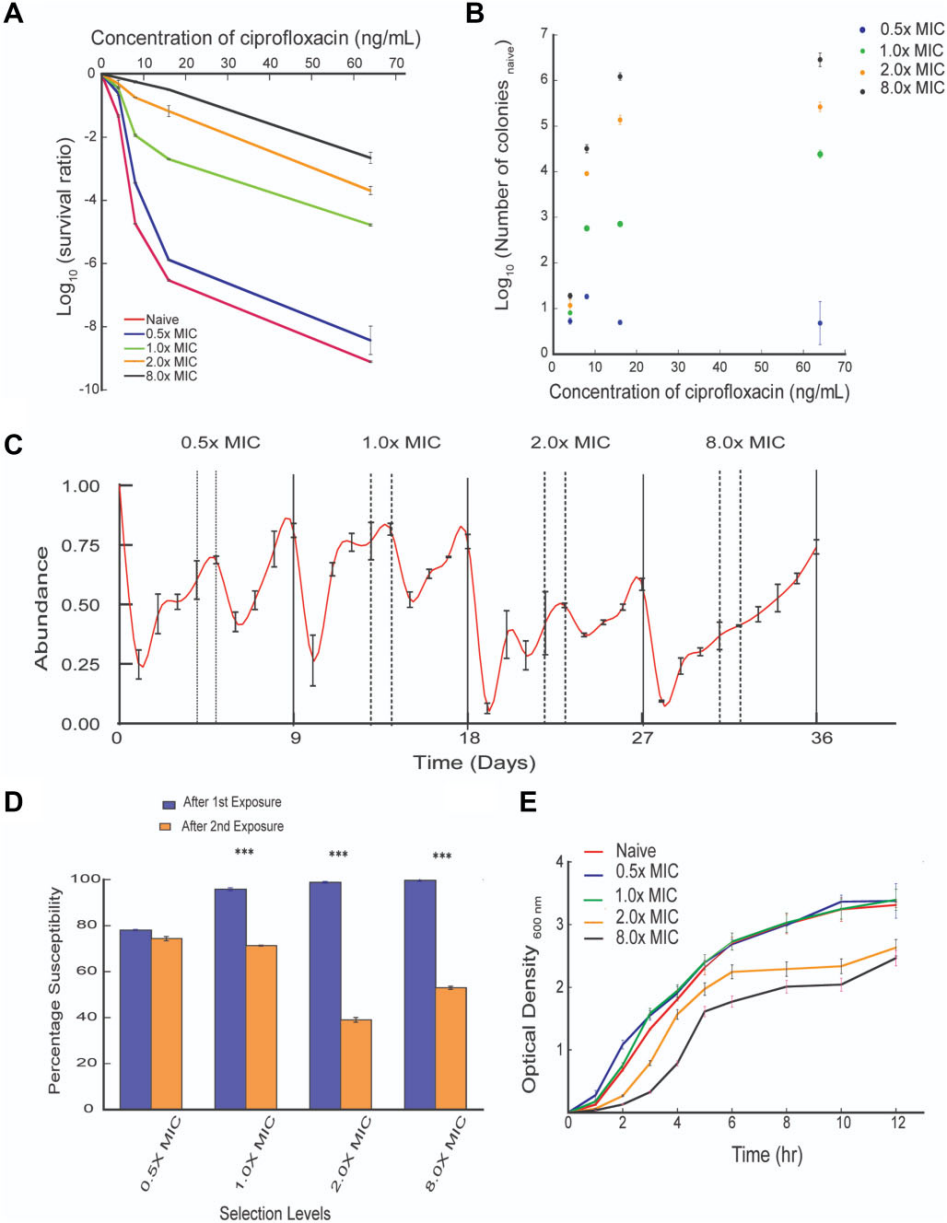
Validation of adaptive-stress response model used for selection of ciprofloxacin-adapted high-fitness subpopulations. (**A**) Survival ratio (ratio of survival of *E. coli* with and without ciprofloxacin pressure) of naïve and evolved *E. coli* subpopulations in Log_10_ scale (Y-axis) are plotted against different ciprofloxacin stress levels (expressed as concentration of ciprofloxacin: X-axis). Upward shifting of survival ratio among ciprofloxacin-adapted subpopulations with respect to the naïve population at different selection levels indicates evolutionary rescue and survival improved progressively with increasing exposure to higher ciprofloxacin gradients. (**B**) Survival of adapted subpopulations is shown in Y-axis as the ratio of CFU (on a log_10_ scale) normalized to that of the naïve population. Different ciprofloxacin concentrations are plotted along X-axis. Increasing distance between the spheres for 0.5x MIC evolved and 8.0X MIC evolved subpopulations with increase in ciprofloxacin pressure indicates more be the selection pressure stronger be the enrichment of the populations with adaptive-resistant subpopulations. (**C**) The changing abundance of surviving subpopulations with time in days indicates typical dynamics of evolutionary rescue. (**D**) After first cycle of ciprofloxacin exposure, the majority of adapted subpopulations revert to susceptibility. However, this plasticity in ciprofloxacin susceptibility diminished significantly (paired *t*-test; *P*< 0.001, indicated by ***) in transfer bottlenecked subpopulations challenged to second cycle of ciprofloxacin exposure. (**E**) Growth kinetics of naïve and the adapted subpopulations.

The minimum inhibitory concentration (MIC) of ciprofloxacin for *E. coli* BW25113 was estimated to be 8ng/mL in our laboratory. The evolutionary rescue was very feeble, and the survival was very low (∼4.42% at 0.5× MIC and <0.001% at higher exposures) for the naïve *E. coli*. However, during transfer bottlenecking with increasing concentrations of ciprofloxacin the evolutionary rescue and survival improved (Figure 1A). For example, re-exposure of 0.5× MIC adapted subpopulation to 0.5× MIC in the second cycle improved survival by about 5-fold (Figure 1A) as compared to that in naïve cells. Interestingly, the survival improved by over 10^6^-fold compared to that of naïve cells at the highest selection level (8.0× MIC; Figure 1A). This finding is consistent with the data calculated for enrichment of population with adapted subpopulations (i.e. total number of surviving cells in adapted strains normalized against naïve population) across selection levels (Figure 1B). When we compared the population enrichment between the highest and the lowest selection levels, we observed that enrichment at 8.0× MIC was several orders of magnitude higher (i.e. 8.0× survived: Log_10_ 5.78 versus 0.5× MIC: Log_10_ 0.55) than that for 0.5× MIC. Furthermore, evolutionary rescue with time upon ciprofloxacin challenge demonstrated typical population dynamics characterized by a rapid killing phase followed by evolutionary rescue (Figure 1C). However, re-exposure of the rescued population after transfer to the same selection level dampened killing and further improved rescue.

At the end of each cycle of ciprofloxacin exposure, the MIC of adapted subpopulations were evaluated using E-strip. The MIC data indicated progressive decrease in ciprofloxacin susceptibility with adaptation to higher concentration of ciprofloxacin (16–23 ng/mL in naïve to 125 ng/ml in subpopulations adapted to 8.0× MIC). However, when they were passaged four times without ciprofloxacin supplementation and re-exposed to the same selection, we observed that >95% of the rescued subpopulations (after first cycle of ciprofloxacin challenge) reverted to a susceptible phenotype (Figure 1D). However, this plasticity in ciprofloxacin susceptibility diminished significantly (Figure 1D, *P*< 0.001) after the transfer-bottleneck. Luria–Delbruck fluctuation assay demonstrated no significant change in colony counts for individual and single cultures, indicating that the mutations were not acquired spontaneously but were induced by ciprofloxacin (Supplementary Figure S2A). Acquisition of mutations during antibiotic stress are known to impart a fitness cost to bacteria (37). Analysis of growth kinetics indicates that adaptation to supra-MIC ciprofloxacin not only prolonged the lag phase (Figure 1E), but also compromised the growth rate and fitness (Supplementary Figure S2B). Taken together, these results indicate that adaptation to increasing concentrations of ciprofloxacin may be associated with antibiotic-induced mutations with a fitness cost. Mutations in canonical targets (e.g. gyrase for ciprofloxacin exposure) are frequently associated with high fitness costs and often require other mutations in non-canonical targets to alleviate fitness (37,38). Interestingly, in our study, the subpopulations adapted to ciprofloxacin had relatively lower fitness costs than those reported for mutations in canonical targets (as the most predominant morphotypes were selected within 16–18 h of plating). These findings suggest that repeated exposures to increasing concentration of ciprofloxacin with transfer bottlenecking facilitate progressive adaptation to the antibiotic through high-fitness mutations in non-canonical targets.

### Identification of non-canonical mutations and the changing genomic landscape during adaptation to ciprofloxacin

**Figure 2.**
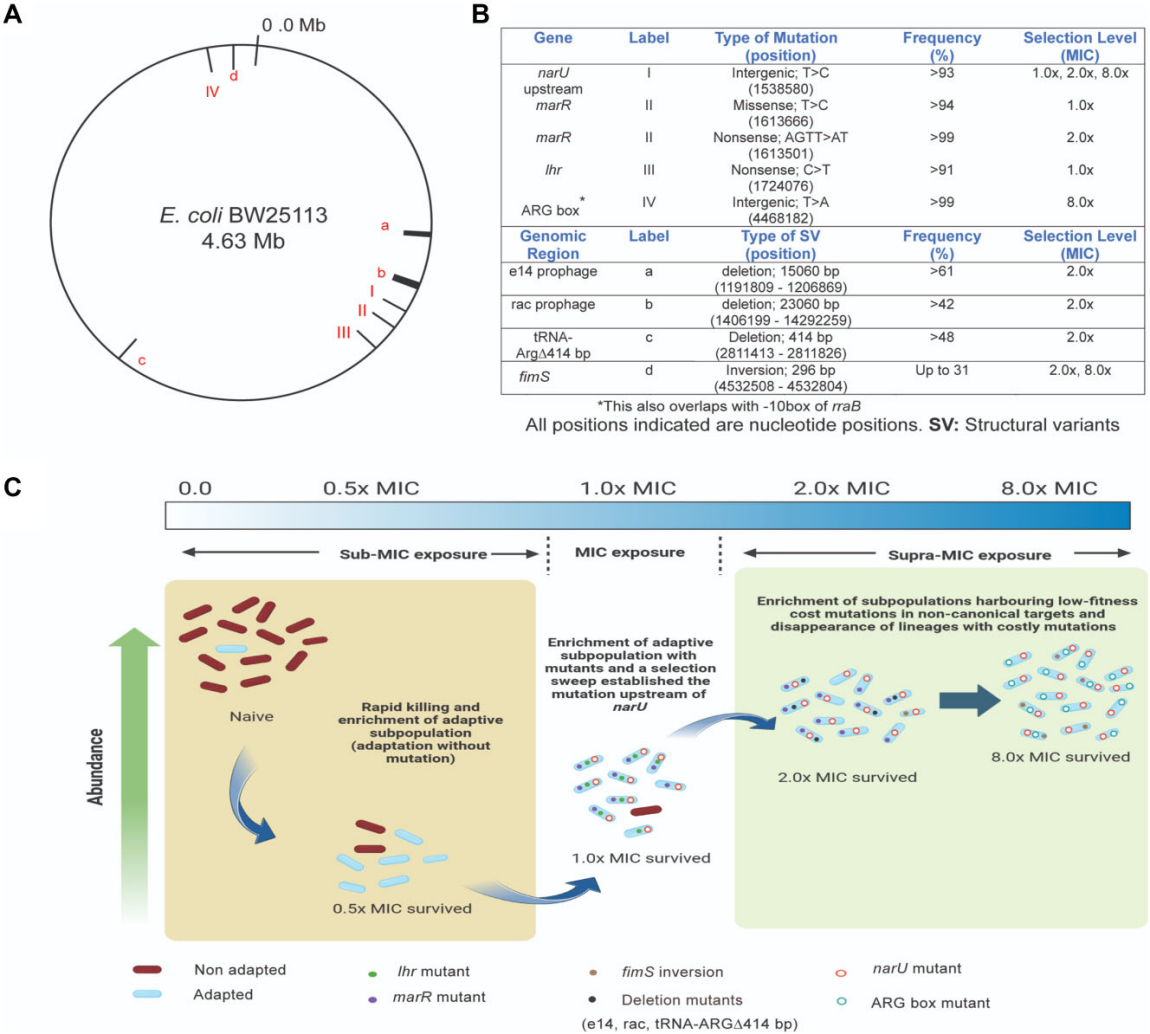
Mutational landscape identified during early adaptation to ciprofloxacin. (**A**, **B**) A map and tabulated list of mutations, small insertion-deletions, and structural variants identified in adapted subpopulations. (**C**) The changing genomic landscape with mutations and structural variants in adapted subpopulations is shown. An intergenic mutation in the regulatory region of narU was consistently present in all adapted subpopulations from 1.0× MIC through 8.0× MIC.

The Oxford Nanopore platform was used for whole genome sequencing of subpopulations adapted to different selection levels. The quality control values related to sequence reads and mapping are shown in Supplementary Table S4. A map of the SNPs, INDELs, and structural variants (SVs) identified in adapted subpopulations is shown in Figure 2A and B. Briefly, we identified a missense mutation (T1613666C; MarR with L97P) and a 2-bp deletion (AGTT1613501A_ _T; truncated MarR) at 1.0× and 2.0× MIC, respectively in the gene body of transcriptional repressor of multiple antibiotic resistance (*marR*; one of the canonical targets of ciprofloxacin stress). The MarR mutations led to the over-expression of *marA* under supra-MIC ciprofloxacin stress (Supplementary Figure S3A). Typically, *marA* positively regulates *acrAB-tolC* which is associated with antibiotic efflux (39). In contrast, here we found overexpression of *acrR*, a local repressor, that led to repression of *acrB* (*P*< 0.05; Supplementary Figure S3B and C). We also found a nonsense mutation (C1724076T; Q245*) at 1.0× MIC within the structural gene of an ATP-dependent helicase superfamily II (*lhr*). Lhr is a DNA repair helicase that has been linked to base-pair melting near the fork branch point (40). However, we did not find evidence of hypermutations in the adapted subpopulations. The *xseA* gene, critical for the repair of ciprofloxacin-induced DNA damage (41), has a ‘marbox’ in the upstream regulatory region (42). Interestingly, we found almost 10-fold higher expression of *xseA* transcripts at both 1.0x and 2.0x MIC levels (*P*< 0.05; Supplementary Figure S3D); this may explain, at least in part, the absence of hypermutations at these selection levels.

**Figure 3.**
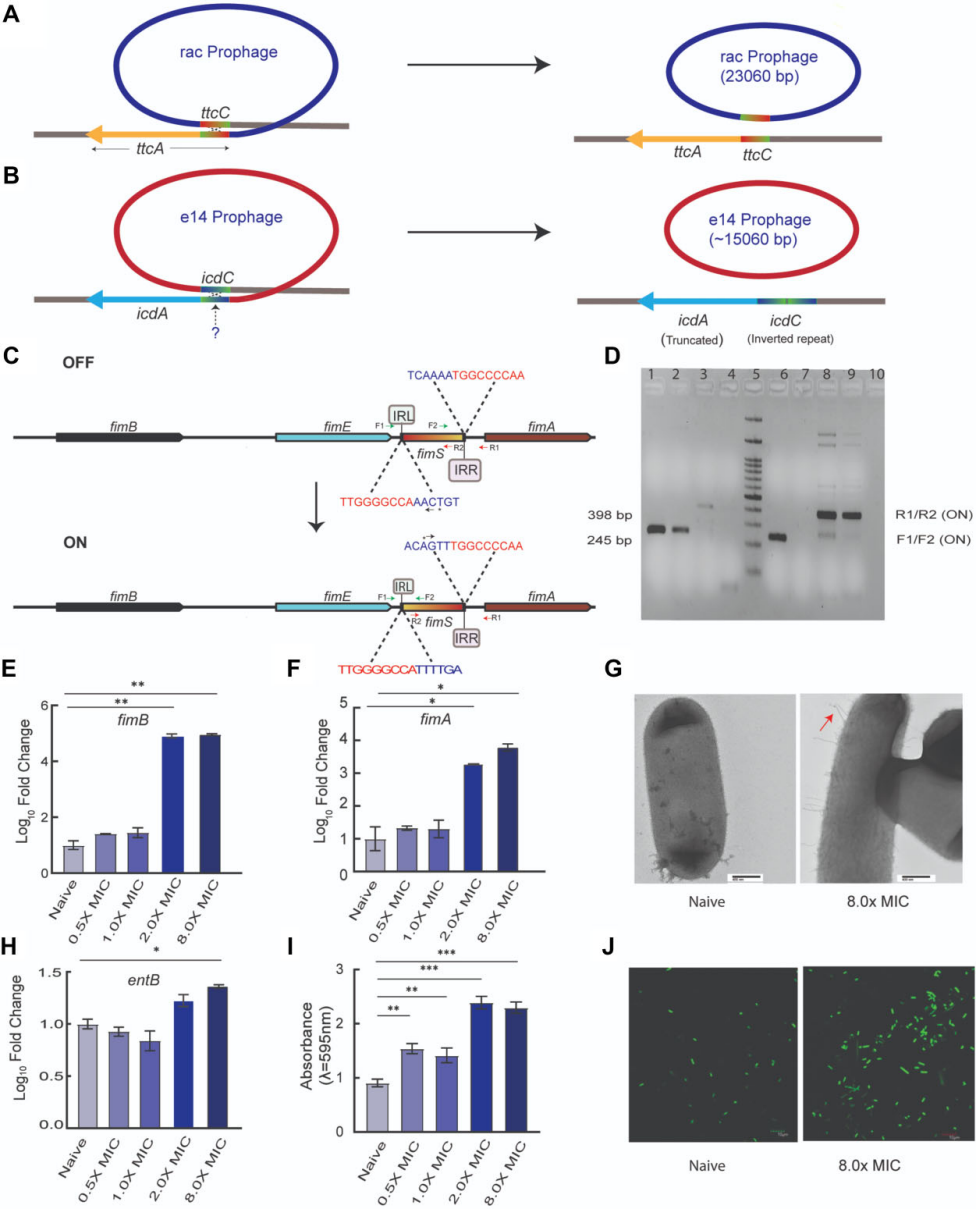
Structural variants linked to fimbriae and biofilm formation at 2.0× and 8.0× MIC. (**A**, **B**) Excision of rac and e14 prophage elements results in mutated tRNA-cytidine (*ttcA*) and isocitrate dehydrogenase (*icdA*). (**C**) Switching-on of fim operon by inversion of fimS promoter region. Green and red coloured arrows indicate forward (F1, F2) and reverse primer (R1, R2) sets for the inversion assay. (fimS: regulatory region of fim operon, *fimB* and *fimE*: recombinase that regulates fim operon's expression, *fimA*: encodes one of the components of type-I fimbriae), IRL: Left inverted repeat, and IRR: right inverted repeat. Nucleotide sequences written in red and blue indicate IRL and IRR, respectively. Asterisk (*) and the overlaid arrow indicates transcription start site and the direction. (**D**) Inversion assay: PCR amplification with F1/F2 and R1/R2 primer sets generate products of 245 and 398 bp respectively. PCR amplification with two forward or reverse primer sets confirmed fimS inversion. Lane 5 exhibited resolved molecular weight marker. The lane numbers of loaded PCR products from 2× survived subpopulation amplified with F1/F2 (Lanes 1, 2), R1/R2 (Lane 3), and F1/R1 (Lane 4) and from 8x survived subpopulation amplified with F1/F2 (Lane 6), F2/R2 (Lane 7), and R1/R2 (Lanes 8, 9) have been mentioned in parentheses after primer sets used. (**E**, **F**) Transcript levels of *fimB* and *fimA* (Log_10_ fold change) estimated for naïve and adapted subpopulations. (**G**) Transmission Electron Microscopic images showing fimbriae formation at 8.0× MIC. (**H**) Transcript levels of enterobactin (*entB*) (Log_10_ fold change) estimated for naïve and adapted subpopulations. (**I**) Crystal Violet Binding Assay indicates enhanced biofilm formation in subpopulations adapted to increasing ciprofloxacin concentration gradients. (**J**) Confocal Laser Scanning Microscopic images demonstrate strong biofilm formation at 8.0× MIC. * *P*< 0.05, ** *P*< 0.005 and *** *P*< 0.001 in paired *t*-test.

Furthermore, adaption to ciprofloxacin led to excision of prophage elements (rac: ∼15 060 bp deletion and e14: 23 060 bp deletion), and the emergence of structural variations (*fimS* phase variation: 296 bp inversion) from 2.0× MIC onwards (Figure 3A–C). The rac excision resulted in mutated tRNA-cytidine(32) 2-sulfurtransferase (*ttcA*) (Figure 3A). In addition, faulty recombination during e14 excision resulted in an inverted repeat within C-terminal domain of isocitrate dehydrogenase (*icdC*) (Figure 3B). Interestingly, structural variant calling without the allelic frequency filter (i.e. allelic frequency filter of >0.3, the default filter for bcftools) identified low frequency (∼5%) *fimS* inversion (phase variation) at 2.0× MIC selection level, which was further validated using the inversion assay (Figure 3C and D). The frequency of this inversion increased to >30% at 8.0× MIC. We also found high transcript levels of *fimB* (recombinase that switches on fim operon) and *fimA* (a component of Type-I fimbriae) supporting phase variation in *fimS* (Figure 3E and F). In addition, elevated enterobactin (*entB*) transcript levels, CV-binding assay, confocal and scanning microscopy suggest that *fimS* inversion may contribute to the induction of fimbrial growth and biofilm formation (Figure 3G–J).

A 414-bp deletion in arginine-carrying tRNA coding gene (tRNA-ArgΔ414-bp) and an intergenic mutation (C4468182T; ‘ARG box’) in between ornithine carbamoyl transferase (*argI*) and inhibitor of RNase E (*rraB*) were identified at 2.0× MIC and 8.0× MIC respectively; neither of these mutations have been reported previously. These mutations are linked to arginine utilization for protein synthesis and arginine catabolism, making them non-canonical targets.

The mutations and structural variants listed in Figure 3B are detectable at specific selection levels (Figure 3B and C). All mutations were detected at > 90% of the adapted subpopulations, while the structural variants were detected in <5–61%, suggesting that latter is more dynamic during adaptation. Interestingly, the intergenic mutation upstream of *narU* (T1538580C) remained consistently detectable from 1.0× MIC through the highest selection level in the backdrop of the changing genomic landscapes. Mutations in *narU* have not been previously linked to antibiotic resistance and it is not recognized as a canonical antibiotic resistance gene. Nonetheless, NarU has been associated with nutritional depletion-associated anaerobiosis (43,44). We, therefore, investigated the biological roles of three non-canonical mutations in metabolism-related genes (i.e. tRNA-ArgΔ414-bp, point mutations in ‘ARG box’ and upstream of *narU*) present in adaptive subpopulations.

### Dysregulation of arginine metabolism and total protein content in adapted subpopulations

**Figure 4.**
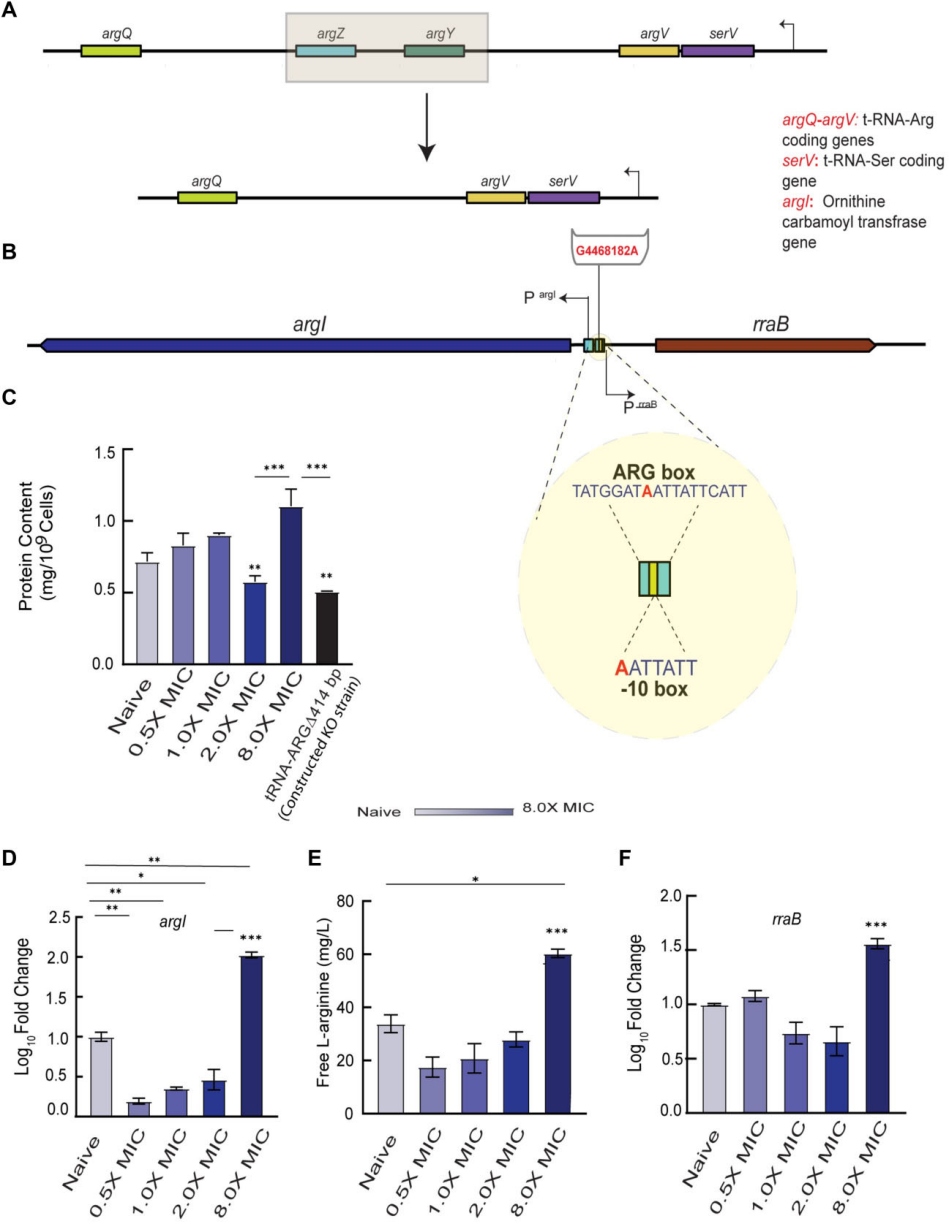
Dysregulation of arginine metabolism and total protein content in subpopulations adapted to supra-MIC ciprofloxacin. (**A**) A genetic map showing a 414-bp deletion in the t-RNA-Arginine coding genes of *E. coli* subpopulations adapted to 2.0× MIC. (**B**) A map showing a point mutation (G4468182A) in the ‘ARG box’ located upstream of ornithine carbamoyl transferase gene (*argI*) that also overlaps with the ‘-10 box’ of RNase E inhibitor coding gene (*rraB*) of *E. coli* subpopulations adapted to 8.0× MIC. (**C**) Total protein content (mg/10^9^ Cells) estimated for naïve, subpopulations adapted to different selection levels and tRNA-ArgΔ414-bp mutant (i.e. constructed KO strain). (**D**) Transcript levels of *argI* (expressed as Log_10_ fold change) estimated for naïve and subpopulations adapted to different selection levels. (**E**) Cytosolic free l-arginine level (mg/l) estimated for naïve and subpopulations adapted to different selection levels. (**F**) Transcript levels of *rraB* (expressed as Log_10_ fold change) estimated for naïve and subpopulations adapted to different selection levels. * *P*< 0.05, ** *P*< 0.005 and *** *P*< 0.001 in paired *t*-test.

The enrichment of positively charged amino acids (e.g. arginine and lysine) at the C-terminal ends of bacterial proteins has been associated with higher protein expression owing to increased half-life^45^. The enrichment of the C-terminal ends with arginine and lysine explains about 85% of variation in protein levels (45). In addition, l-arginine levels in bacteria have been linked to stress response to antibiotic tolerance (46); however, the underlying mechanisms are poorly understood. We therefore investigated the impact of tRNA-ArgΔ414-bp and the mutation at the overlapping promoters of ornithine carbamoyl transferase (*argI*) and RNaseE inhibitor (*rraB*) disrupting the ‘ARG box’ (*argI*) and the ‘-10 box’(*rraB*) (Figure 4A and B). The tRNA-ArgΔ414-bp was detected only at 2.0× MIC adapted subpopulations and interestingly, the total protein levels for this subpopulation were the lowest compared to that in the naïve and other adapted subpopulations (Figure 4C). We have also constructed tRNA-ArgΔ414-bp knock-out mutant *E. coli*. The total protein content was significantly lower in both the 2.0× MIC adapted *E. coli* (containing the tRNA-ArgΔ414-bp) and the tRNA-ArgΔ414-bp knock-out mutant *E. coli* (0.576 mg/10^9^ cells in 2.0× MIC adapted and 0.508 mg/10^9^ cells in the KO construct) as compared to the naïve *E. coli* (0.716 mg/10^9^ cells) (Figure 4C). This finding confirming that the tRNA-ArgΔ414 affects total protein content in bacteria. The binding of argR to ‘ARG box’ negatively regulates *argI* expression (47). Ornithine carbamoyl transferase (*argI*) is a key enzyme for arginine biosynthesis from ornithine (48). Here, we found that the 8.0x MIC adapted subpopulations with the ‘ARG box’ mutation had significantly higher levels (over 7-fold higher) of *argI* expression compared to that in naïve and other adapted subpopulations (*P*< 0.001; Figure 4D). The mutated promoter had significantly higher promoter activity as compared to the wildtype promoter (*P*< 0.05) (Supplementary Figure S4), indicating that the ‘ARG box’ mutation may facilitate higher *argI* transcript level. The discrepancy in between promoter activity and mRNA transcript level of *argI* can be explained in part by autogenous regulation of arginine biosynthesis pathway (49). Furthermore, l-arginine levels were significantly elevated in the 8.0× MIC adapted compared to the naïve and other adapted subpopulations (*P*< 0.001; Figure 4E). This finding suggests that the ‘ARG box’ mutation leads to increased *argI* levels resulting in increased l-arginine levels.

Since the ‘ARG box’ mutation overlapped with the -10 box of *rraB*, we also analysed *rraB* transcripts. We found that the ‘ARG box’ mutation in the 8.0x MIC adapted subpopulations was associated with significantly higher levels of *rraB* (over 6-fold higher) than that in naïve and other adapted subpopulations (*P*< 0.001; Figure 4F). RraB inhibits RNase E (an endonuclease) that regulates mRNA decay, rRNA and tRNA maturation (50,51). Of note, the 8.0× MIC-adapted subpopulations showed the highest level of total proteins (Figure 4C). We argue that the ‘ARG box’ mutation at –10 box of *rraB* occurring at 8.0× MIC adapted subpopulations is located at a strategic genomic location that may directly regulate mRNA stability and maturation (through *rraB* levels) and protein stability (through arginine levels) in bacteria. Thus the ‘ARG box’ mutation may explain the highest level of total proteins at 8.0× MIC levels (Figure 4C).

### NarU facilitates shifting to anaerobiosis and early adaption to ciprofloxacin stress

**Figure 5.**
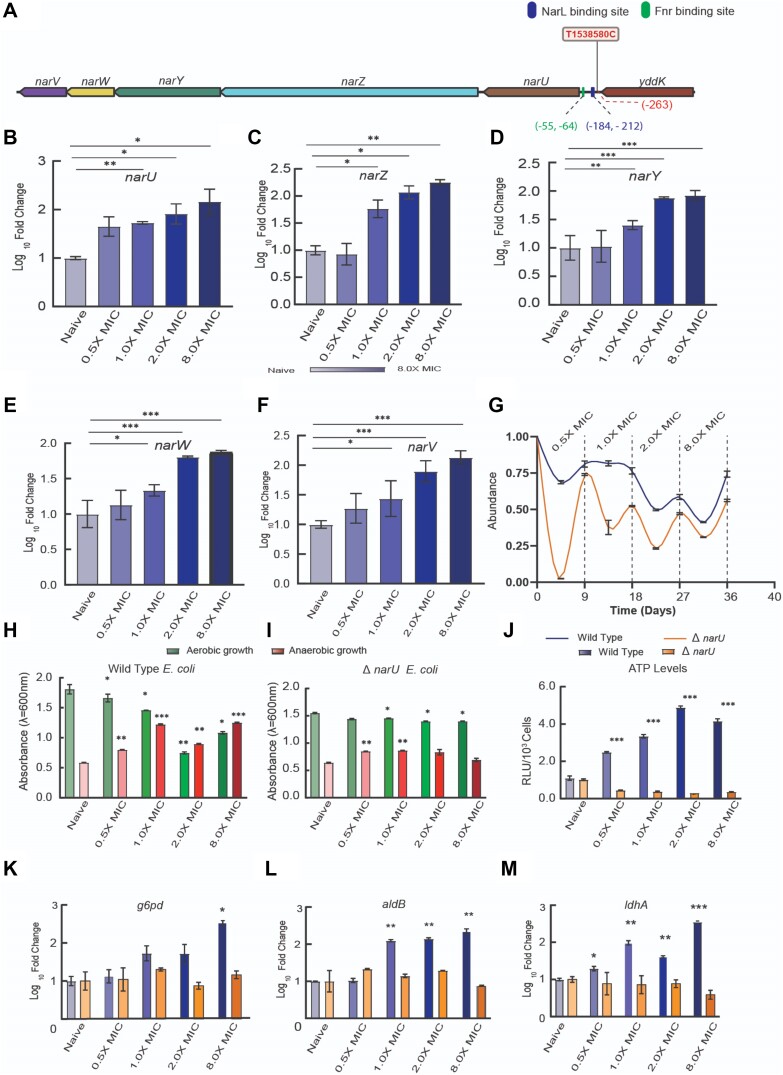
Role of *narUZYWV* operon in anaerobiosis, early adaptation to ciprofloxacin stress, and increased ATP production. (**A**) A map showing an intergenic mutation in the regulatory region of *narU* consistently detected in adapted subpopulations from 1.0× through 8.0× MIC. (**B–F**) Log_10_ fold change of *narU*, *narZ*, *narY*, *narW* and *narV* transcript levels in naïve and adapted subpopulations. (**G**) The evolutionary rescue was feeble in *narU* knockout (Δ*narU*) *E. coli* when compared to that of wild type *E. coli* suggesting a putative role for *narU* in early adaptation to ciprofloxacin stress. (H, I) Growth (i.e. OD_600_) of naïve and adapted wild type (**H**) and Δ*narU* strains (**I**) under aerobic and microaerophilic (low oxygen concentrations) conditions respectively. (**J**) ATP production (measured as Relative Luminescence Unit i.e. RLU/10^3^cell) estimated for naïve and adapted subpopulations of wild type and Δ*narU strains*. (**K–M**) Transcript levels of glucose-6-phosphate dehydrogenase (*g6pd*), aldehyde dehydrogenase (*aldB*), and lactate dehydrogenase (*ldhA*) for naïve and subpopulations adapted to different selection levels. * *P*< 0.05, **P< 0.005 and *** *P*< 0.001 in paired *t*-test.

We identified a stable intergenic mutation (T1538580C) upstream to *narU* that was consistently detected from 1.0× MIC through 8.0× MIC (Figure 5A). This mutation in the putative regulatory region of *narUZYWV* operon was associated with significantly higher levels of *narU*, *narZ*, *narY*, *narW* and *narV* expression compared to that in the naïve population (*P*< 0.05; Figure 5B–F). The mutated *narU* regulatory region construct was associated with significantly higher promoter activity compared to that of the wild type construct (*P*< 0.05) (Supplementary Figure S4), confirming a role for the mutation in the *narU* upstream element in regulating the *narUZYWV* operon. Similar to arginine biosynthesis pathway, autogenous regulation of *narUZYWV* operon (52) partly explains why the increase in promoter activity may not be proportion to the increase observed in the transcript level of nar genes.

High *narU* levels have been linked to anaerobic growth under severe nutritional stress, thermotolerance, and acid tolerance (43,44). Nonetheless, specific mutations leading to increased expression of *narU* have not been reported. Further, neither *narU* mutations nor over-expression of *narU* have been associated with antibiotic stress previously. To better understand the role of this mutation upstream of *narU*, we studied a *narU* knockout (Δ*narU* strain). Our results show that the evolutionary rescue in the Δ*narU* strain is feeble compared to that in the wildtype at all selection levels (Figure 5G), suggesting a role for *narU* in evolutionary rescue during ciprofloxacin stress.

Subpopulations adapted at 1.0×, 2.0× and 8.0× MIC expressing higher levels of the transcripts of *narUZYWV* operon exhibited significantly improved growth under microaerophilic conditions (Figure 5H) compared to the naïve population (with basal level *narUZYWV* operon transcripts). However, for the Δ*narU* strain, growth under microaerophilic conditions was comparable between the naïve and the supra-MIC adapted subpopulations (Figure 5I), further strengthening the role of *narU* in anaerobiosis during adaptation to ciprofloxacin. Anaerobic growth in bacteria has been linked to higher ATP levels (53). In addition, the naïve populations of the wild-type and the Δ*narU* strain had comparable levels of ATP production (Figure 5J). However, the adapted wild-type subpopulations produced up to 10-fold higher levels of ATP compared to that by the Δ*narU* strain (*P*< 0.001; Figure 5J), indicating a critical role of NarU in meeting the high ATP demand in anaerobic growth conditions.

We then measured the activities of rate-limiting enzymes in aerobic respiration. The activity of Isocitrate dehydrogenase (IcdA), the rate-limiting enzyme of TCA cycle, was comparable among naïve and adapted subpopulations for both the wild-type and the Δ*narU* strain (Supplementary Figure S5A), suggesting that NarU expression may not affect the basal energy flux through aerobic metabolism.

Glucose-6-phosphate dehydrogenase (*g6pd*), aldehyde dehydrogenase (*aldB*) and lactate dehydrogenase (*ldhA*) are the rate limiting enzymes for pentose phosphate, alcohol and lactatic acid fermentation respectively. The *g6pd*, *aldB* and *ldhA* transcript levels showed an increasing trend with adaptation to increasing concentrations of ciprofloxacin for wild-type *E. coli* (Figure 5K–M). In contrast, for the Δ*narU* strain, *g6pd*, *aldB* and *ldhA* transcript levels were comparable for the naïve and all adapted subpopulations; this finding reiterates a role for NarU in anaerobiosis under antibiotic stress.

L-arginine can be catabolized to produce ATP under anaerobic conditions through the ADI (Arginine deiminase) pathway (46). Intrigued by mutations linked to arginine utilization and biosynthesis in subpopulations adapted to 2.0×- and 8.0×-MIC, we sought to understand further the link between high ATP levels and arginine metabolism. Hence, we estimated the transcript levels of arginine succinyltransferase (*astA*) and arginine deiminase (*arcA*) which are the rate limiting enzymes for arginine catabolism under aerobic and anaerobic conditions respectively. We found comparable levels of *astA* in the naïve and subpopulations adapted to 2.0× and 8.0× MIC (Supplementary Figure S5B), ruling out a role for aerobic arginine catabolism during adaptation. The *arcA* transcript levels were significantly higher (*P*< 0.005; Supplementary Figure S5C) for subpopulations adapted to 2.0× and 8.0× MIC compared to the naïve population, suggesting that arginine catabolism through the ADI pathway contributes to ATP production during adaption to ciprofloxacin. In addition, the ‘ARG box’ mutation in the 8.0x MIC adapted subpopulations led to high L-arginine levels, which could directly contribute to increased ATP synthesis through the ADI pathway. Taken together, our findings highlight the interplay between two key mutations (i.e. Mutation upstream of *narU* and in the ‘ARG box’), anaerobiosis and ATP levels in early adaptation to ciprofloxacin stress.

### Mutations in the regulatory regions of *narU* and *argI* (‘ARG box’) are clinically relevant

We found that over 70% of *E. coli* whole-genome sequences (*n* = 661) from Pathosystems Resource Integration Center (PATRIC) and *E. coli* whole genome sequences we had reported earlier (*n* = 89) and those isolated from gut of healthy children (*n* = 113) harboured mutations in the regulatory of *narU* and *argI* (Supplementary Figure S6A–C). Interestingly, mutations in the *narU* or the *argI* regulatory regions were more frequently detected in *E. coli* isolates phenotypically resistant to ciprofloxacin compared to their susceptible counterparts (Supplementary Figure S6D; *P*< 0.05). Furthermore, mutations in the intergenic regions of *narU* and *argI* co-occurred in ∼43% (38/89) of clinical isolates (Supplementary Figure S6D) of which 94.7% were resistant to ciprofloxacin.

## Discussion

Evolutionary rescue, the most prominent strategy for bacteria to evade antibiotics (16), leads to selection of high fitness mutants through clonal interference in the adapted subpopulations. While evolutionary rescue during antibiotic stress is well-documented, the underlying mechanisms remain poorly understood. Here, we used transfer bottlenecking to capture high-fitness mutant lineages during early adaptation to ciprofloxacin stress. We sought to identify non-canonical mutations in early adaptation and investigate their role in evolutionary rescue. The bottlenecking method used does not favour the selection of mutations in canonical targets owing to their high fitness cost.

In total, we found five mutations and four structural variants in adapted subpopulations (Figure 2B). Interestingly, a previously unrecognized mutation in the regulatory region of *narU* appeared as the constant backbone of the changing mutational landscape at various selection levels. The rate of back mutation reported for *E. coli* (>10^−10^ mutations per nucleotide) is much lower than the frequency of loss of mutant lineages from the adapting population (54). In addition, clonal interference can outcompete beneficial lineage with a fitter mutant lineage (55) at a higher selection level. Hence, we believe that the transient nature of most of the mutations (except for the mutation in the regulatory region of *narU*) at different selection levels may be due to progressive selection of the fittest lineage rather than back mutations. We have found that *marR, lhr* mutant lineage that appeared in 1.0× and 2.0× MIC were outcompeted by strong biofilm forming lineages harbouring mutation in ‘ARG Box’ and the fimS inversion at 8.0× MIC. Although, excision of prophage elements (e14, rac) at 2.0× MIC may have contributed to the induction of biofilm formation, this lineage was deselected at 8.0× MIC, with the appearance of fimS inversion (allows fimbrial growth that facilitates maturation of biofilms; Figure 3C–G) and the ‘ARG box’ mutation (allows increased synthesis of arginine for biofilms; Figure 4B, E). Interestingly, about 33% (i.e. 64/193) ciprofloxacin-resistant clinical strains of *E. coli* exhibited fimS inversion. This finding suggests that fimS inversion is more common among ciprofloxacin-resistant clinical *E. coli* isolates than previously recognized. In other words, the presence of fimS inversion among clinical *E. coli* isolates may be linked to ciprofloxacin-adaptation or resistance.

We further investigate the biological role of three of the mutations we identified were linked to metabolism: (a) tRNA-ArgΔ414-bp (b) ‘ARG box’ mutation and (c) mutation in the regulatory region of *narU*. Previous studies demonstrated small deletion and point mutations within tRNA-Val/-Lys/-Ala to be essential for the genomic evolution of antibiotic resistance (56,57). Our results indicate that the tRNA-ArgΔ414-bp at 2.0× MIC restricts l-arginine utilization in protein synthesis as evidenced by significantly lower total protein levels (Figure 4C). Despite reduced utilization for protein synthesis, l-arginine levels were lower in the adapted subpopulations with the tRNA-ArgΔ414-bp, indicating its utilization for other purposes. Of note, the adapted subpopulations with tRNA-ArgΔ414-bp had the highest levels of both ATP (Figure 5J) and *arcA* transcripts (Supplementary Figure S5C), indicating the utilization of l-arginine for energy production via the ADI pathway. In addition, in the adapted subpopulation with the tRNA-ArgΔ414-bp, we found about higher biofilm formation and higher levels of *entB* (a siderophore induced by l-arginine metabolism that induces biofilm formation) (58). Taken together, the tRNA-ArgΔ414-bp enables increased ATP production and increased biofilm formation at the cost of reduced protein synthesis. Along with the tRNA-ArgΔ414-bp at 2.0x MIC, we also identified several structural variants (i.e. excision of e14 and rac, and *fimS* inversion) that have been reported to enhance biofilm formation (59,60). We believe that the deselection of the tRNA-ArgΔ414-bp at 8.0× MIC (Figure 3B) may relieve the constraints on global protein synthesis. Interestingly, at 8.0× MIC, the ‘ARG box’ mutation upstream of *argI* that overlaps with the -10 box (Pribnow box) of *rraB*, which stabilizes RNA metabolism leading to increased total protein content (Figure 4B and C). Mutations reported in the Pribnow box are limited to a few antibiotic-resistant genes (ARGs) associated with amoxicillin and β-lactam resistance (61,62). The ‘ARG box’ mutation in 8.0x MIC adapted subpopulations led to increased levels of *argI* and *rraB*, which in turn results in the highest level of total protein as well as L-arginine at this selection level (Figure 4C and E).

A strong selective sweep established a mutation at the upstream region of *narU* that was detected consistently in adapted subpopulations from 1.0× through 8.0× MIC. This mutation was associated with elevated level of *narUZYWV* transcripts, resulting in anaerobiosis and significantly higher ATP levels (Figure 5J). The *narU* encodes a nitrate/nitrite transporter, while *narZYWV* codes for nitrate reductase Z (NRZ) (63). Although, the physiological role of this operon remains poorly understood, previous studies suggest that both NarU and NRZ help the bacteria to adapt anaerobic metabolism in the transition to anaerobiosis under atypical conditions such as severe nutritional stress, thermal and acid exposure (43,44,63). However, to the best of our knowledge, there is no documented literature linking antibiotic resistance to mutation in the regulatory region of *narUZYWV* operon. It is noteworthy that our results with the Δ*narU* strain indicate a previously unrecognized role for *narUZYWV* operon, in particular, the role of NarU, in evolutionary rescue, anaerobiosis and ATP production during antibiotic stress (Figure 5G–J). While *narUZYWV* operon has been demonstrated to induce anaerobiosis; *argI* can regulate arginine biosynthesis, facilitating energy metabolism through the ADI pathway under oxygen-limiting conditions (46). Hence, we speculate that the consistent intergenic mutation upstream of *narU* provides the necessary platform for the ARG box mutant lineage to thrive better under anaerobic conditions. Our data reveals a previously unrecognized role for *narU*-mediated adaptive responses to ciprofloxacin stress.

Decades of consistent efforts have helped investigate the role of antibiotic-resistant genes (ARGs) in antimicrobial resistance (AMR) development. However, only in the last few years there has been a spike in research focussing in understanding the contribution of metabolic genes to AMR. A recent paper has probed clinical strains to identify metabolism-specific mutations and demonstrated the role of a representative mutation in the 2-oxoglutarate dehydrogenase (*sucA*) enzyme in lowering basal respiration through the TCA cycle and giving rise to antibiotic resistance (12). Here, we have analyzed ∼875 whole genome sequences of clinical strains of *E*.*coli* and estimated ∼70% of them to harbour at least one of the mutations at upstream region of *narU* and *argI*. Interestingly, approximately 12% of ciprofloxacin-resistant clinical isolates of *E. coli* carried mutations at the exact same positions (G4468182A within the ‘ARG box’ or T1538580C within the ‘narU upstream element’) as those found in the laboratory-adapted strain *E. coli* BW25113. Further, our study highlights how mutations in the regulatory region of these metabolic genes facilitate early adaptation to ciprofloxacin stress. However, we cannot ascertain whether these adaptive responses are specific to ciprofloxacin or fluoroquinolone. We cannot rule out if the stimulation of *narU*-mediated adaptive response overlaps with a general stress response or response to other antibiotics in bacteria.

Specific mutations associated with increased ATP production during antibiotic stress have not been previously recognized. Our data suggests that bacteria may use a multipronged and coordinated genetic approach to facilitate biofilm formation, anaerobiosis and high ATP production during early adaptation. Specific mutations (tRNA-ArgΔ414-bp, ‘ARG box’ mutation and the mutation upstream of *narU*) linked to nitrogen and carbon metabolism appear to be the central theme for physiological changes necessary for rescue under ciprofloxacin stress. Furthermore, the presence of mutations in the regulatory regions of *narU* and *argI* among ciprofloxacin resistant *E. coli* corroborates their clinical relevance.

This work suggests that metabolic reprogramming during adaptation to antibiotics involves switching to anaerobic metabolism and increased ATP production through mutations in non-coding regions. Our findings emphasize the importance of analysing mutations in regulatory regions of metabolic genes during adaption to antibiotic stress and the emergence of AMR.

## Supplementary Material

gkae719_Supplemental_Files

## Data Availability

All sequencing data are available on the Sequence Read Archive repository under BioProject accession no. PRJNA931653, PRJNA942108, PRJNA794291, PRJNA634509, PRJNA634478. All the accession numbers of the Sanger sequencing data (Mutations in adapted and the corresponding wild type sequences in Naive) are available in Supplementary Table S5.

## References

[1] Shariati A. , Arshadi M., Khosrojerdi M.A., Abedinzadeh M., Ganjalishahi M., Maleki A., Heidary M., Khoshnood S. The resistance mechanisms of bacteria against ciprofloxacin and new approaches for enhancing the efficacy of this antibiotic. Front. Public Health. 2022; 10:1025633. 36620240 10.3389/fpubh.2022.1025633 PMC9815622

[2] Neyestani Z. , Khademi F., Teimourpour R., Amani M., Arzanlou M. Prevalence and mechanisms of ciprofloxacin resistance in *Escherichia coli* isolated from hospitalized patients, healthy carriers, and wastewaters in Iran. BMC Microbiol. 2023; 23:191. 37460988 10.1186/s12866-023-02940-8 PMC10351176

[3] Basu S. , Mukherjee M. Conjugal transfer of PMQR from uropathogenic *E. coli* under high ciprofloxacin selection pressure generates gyrA mutation. Microb. Pathog. 2019; 132:26–29. 30999022 10.1016/j.micpath.2019.04.021

[4] Zlamal J.E. , Leyn S.A., Iyer M., Elane M.L., Wong N.A., Wamsley J.W., Vercruysse M., Garcia-Alcalde F., Osterman A.L. Shared and unique evolutionary trajectories to ciprofloxacin resistance in gram-negative bacterial pathogens. mBio. 2021; 12:e0098721. 34154405 10.1128/mBio.00987-21 PMC8262867

[5] Fernandez L. , Hancock R.E. Adaptive and mutational resistance: role of porins and efflux pumps in drug resistance. Clin. Microbiol. Rev. 2012; 25:661–681. 23034325 10.1128/CMR.00043-12 PMC3485749

[6] Carja O. , Plotkin J.B. Evolutionary rescue through partly heritable phenotypic variability. Genetics. 2019; 211:977–988. 30696715 10.1534/genetics.118.301758 PMC6404248

[7] Dawan J. , Ahn J. Bacterial stress responses as potential targets in overcoming antibiotic resistance. Microorganisms. 2022; 10:1385. 35889104 10.3390/microorganisms10071385 PMC9322497

[8] Pribis J.P. , Garcia-Villada L., Zhai Y., Lewin-Epstain O., Wang A.Z., Liu J., Xia J., Mei Q., Fitzgerald D.M., Bos. J. et al. Gamblers: an antibiotic-induced evolvable cell subpopulation differentiated by Reactive-oxygen-induced general stress response. Mol. Cell. 2019; 74:785–800. 30948267 10.1016/j.molcel.2019.02.037 PMC6553487

[9] Miller K. , O’Neill A.J., Chopra I. Response of *Escherichia coli* hypermutators to selection pressure with antimicrobial agents from different classes. J. Antimicrob. Chemother. 2002; 49:925–934. 12039884 10.1093/jac/dkf044

[10] Bie L. , Zhang M., Wang J., Fang M., Li L., Xu H., Wang M. Comparative analysis of transcriptomic response of *Escherichia coli* K-12 MG1655 to nine representative classes of antibiotics. Microbiol. Spectr. 2023; 11:e0031723. 36853057 10.1128/spectrum.00317-23 PMC10100721

[11] Gottesman S. Trouble is coming: signaling pathways that regulate general stress responses in bacteria. J. Biol. Chem. 2019; 294:11685–11700. 31197038 10.1074/jbc.REV119.005593 PMC6682744

[12] Lopatkin A.J. , Bening S.C., Manson A.L., Stokes J.M., Kohanski M.A., Badran A.H., Earl A.M., Cheney N.J., Yang J.H., Collins J.J. Clinically relevant mutations in core metabolic genes confer antibiotic resistance. Science. 2021; 371:eaba0862. 33602825 10.1126/science.aba0862 PMC8285040

[13] Garoff L. , Pietsch F., Huseby D.L., Lilja T., Hughes. D Population bottlenecks strongly influence the evolutionary trajectory to fluoroquinolone resistance in *Escherichia coli*. Mol. Biol. Evol. 2020; 37:1637–1646. 32031639 10.1093/molbev/msaa032 PMC7253196

[14] CLSI. Performance Standards for Antimicrobial Susceptibility Testing CLSI Supplement M100. 2020; 30th edn. Wayne, PA, USA Clinical and Laboratory Standards Institute.

[15] Suarez S.A. , Martiny A.C. Gene amplification uncovers large previously unrecognized cryptic antibiotic resistance potentialin *E. coli*. Microbiol Spectr. 2021; 9:e0028921. 34756069 10.1128/Spectrum.00289-21 PMC8579933

[16] Zhou D.H. , Zhang Q.G. Compensatory adaptation and diversification subsequent to evolutionary rescue in a model adaptive radiation. Ecol. Evol. 2021; 11:9689–9696. 34306654 10.1002/ece3.7792 PMC8293784

[17] Adam M. , Murali B., Glenn N.O., Potter S.S. Epigenetic inheritance-based evolution of antibiotic resistance in bacteria. BMC Evol. Biol. 2008; 8:52. 18282299 10.1186/1471-2148-8-52 PMC2262874

[18] Wu D. , Ding Y., Yao K., Gao W., Wang Y. Antimicrobial resistance analysis of clinical *Escherichia coli* isolates in neonatal ward. Front Pediatr. 2021; 9:670470. 34113589 10.3389/fped.2021.670470 PMC8185016

[19] Sanchez-Romero M.A. , Casadesus J. Contribution of phenotypic heterogeneity to adaptive antibiotic resistance. Proc. Natl. Acad. Sci. U.S.A. 2012; 111:355–360. 10.1073/pnas.1316084111 PMC3890857 24351930

[20] Durso L.M. , Smith D., Hutkins R.W. Measurements of fitness and competition in commensal *Escherichia coli* and *E. coli* O157:H7 strains. Appl. Environ. Microb. 2004; 70:6466–6472. 10.1128/AEM.70.11.6466-6472.2004 PMC525243 15528507

[21] De Coster W. , D’Hert S., Schultz D.T., Cruts M., Van Broeckhoven C NanoPack: visualizing and processing long-read sequencing data. Bioinformatics. 2018; 34:2666–2669. 29547981 10.1093/bioinformatics/bty149 PMC6061794

[22] Li H. Minimap2: pairwise alignment for nucleotide sequences. Bioinformatics. 2018; 34:3094–3100. 29750242 10.1093/bioinformatics/bty191 PMC6137996

[23] Li H. , Handsaker B., Wysoker A., Fennell T., Ruen J., Homer N., Marth G., Abecasis G., Durbin R. 1000 Genome Project Data Processing Subgroup The sequence alignment/map format and SAMtools. Bioinformatics. 2009; 25:2078–2079. 19505943 10.1093/bioinformatics/btp352 PMC2723002

[24] Sedlazeck F.J. , Rescheneder P., Smolka M., Fang H., Nattestad M., Haeseler A.V., Schatz M.C. Accurate detection of complex structural variations using single-molecule sequencing. Nat. Methods. 2018; 15:461–468. 29713083 10.1038/s41592-018-0001-7 PMC5990442

[25] Danecek P. , Bonfield J.K., Liddle J., Marshall J., Ohan V., Pollard M.O., Whitwham A., Keane T., McCarthy S.A., Davis R.M. et al. Twelve years of SAMtools and BCFtools. Gigascience. 2021; 10:giab008. 33590861 10.1093/gigascience/giab008 PMC7931819

[26] Saldaña-Ahuactzi Z. , Soria-Bustos J., Martínez-Santos V.I., Yañez-Santos J.A., Martínez-Laguna Y., Cedillo-Ramirez M.L., Puente J.L., Girón J.A. The Fis nucleoid protein negatively regulates the phase variation *fimS* switch of the type 1 pilus operon in enteropathogenic *Escherichia coli*. Front. Microbiol. 2022; 13:882563. 35572706 10.3389/fmicb.2022.882563 PMC9096935

[27] Karunakaran. R. , Mauchline T.H., Hosie A.H., Poole P.S A family of promoter probe vectors incorporating autofluorescent and chromogenic reporter proteins for studying gene expression in gram-negative bacteria. Microbiol. 2005; 151:3249–3256. 10.1099/mic.0.28311-0 16207908

[28] Maiti P.K. , Halder J., Mukherjee P., Dey R. Anaerobic culture on growth efficient bi-layered culture plate in a modified candle jar using a rapid and slow combustion system. Indian J. Med. Microbiol. 2013; 31:173–176. 23867675 10.4103/0255-0857.115218

[29] Strobel H.J. Basic laboratory culture methods for anaerobic bacteria. Methods Mol. Biol. 2009; 581:247–261. 19768627 10.1007/978-1-60761-214-8_16

[30] O’Toole G.A. Microtiter dish biofilm formation assay. J. Vis. Exp. 2011; 30:2437. 10.3791/2437 PMC3182663 21307833

[31] Grossman A.B. , Burgin D.J., Rice K.C. Quantification of *Staphylococcus aureus* biofilm formation by Crystal violet and confocal microscopy. Methods Mol. Biol. 2021; 2341:69–78. 34264462 10.1007/978-1-0716-1550-8_9

[32] Burt S.A. , Adolfse S.J.M., Ahad D.S.A., Tersteeg-Zijderveld M.H.G., Jongerius-Gortemaker B.G.M., Post J.A., Brüggemann H., Santos R.R. Cinnamaldehyde, carvacrol and organic acids affect gene expression of selected oxidative stress and inflammation markers in IPEC-J2 cells exposed to *Salmonella typhimurium*. Phytother. Res. 2016; 30:1988–2000. 27561686 10.1002/ptr.5705 PMC5157771

[33] Datensko K.A. , Wanner B.L. One-step inactivation of chromosomal genes in *Escherichia coli* K-12 using PCR products. Proc. Natl. Acad. Sci. U.S.A. 2000; 97:6640–6645. 10829079 10.1073/pnas.120163297 PMC18686

[34] Devanga Raghupathi N.K. , Veeraraghavan B., Sethuvel D.P.M., Anandan S., Vasudevan K., Neeravi A.R., Daniel J.L.K., Sathyendra S., Iyadurai R., Mutreja A. First Indian report on genome-wide comparison of multidrug-resistant *Escherichia coli* from blood stream infections. PLoS One. 2020; 15:e0220428. 32101543 10.1371/journal.pone.0220428 PMC7043739

[35] Zhao Q. , Shen Y., Chen G., Luo Y., Cui S., Tian Y Prevalence and molecular characterization of fluoroquinolone-resistant *Escherichia coli* in healthy children. Front. Cell. Infect. Microbiol. 2021; 11:743390. 34966693 10.3389/fcimb.2021.743390 PMC8710580

[36] Catoiu E.A. , Phaneuf P., Monk J., Palsson B.O. Whole-genome sequences from wild-type and laboratory-evolved strains define the alleleome and establish its hallmarks. Proc. Natl. Acad. Sci. U.S.A. 2023; 120:e2218835120. 37011218 10.1073/pnas.2218835120 PMC10104531

[37] Melnyk A.H. , Wong A., Kassen R. The fitness costs of antibiotic resistance mutations. Evol. Appl. 2015; 8:273–283. 25861385 10.1111/eva.12196 PMC4380921

[38] Pi R. , Liu Q., Takiff H.E., Gao Q. Fitness cost and compensatory eolution in levofloxacin-resistant *mycobacterium aurum*. Antimicrob. Agents Chemother. 2020; 64:e00224-20. 32482677 10.1128/AAC.00224-20 PMC7526816

[39] Weston N. , Sharma P., Ricci V., Piddock L.J. Regulation of the AcrAB-TolC efflux pump in *Enterobacteriacea*e. Res. Microbiol. 2018; 169:425–431. 29128373 10.1016/j.resmic.2017.10.005

[40] Buckley R.J. , Kramm K., Cooper C.D.O., Grohmann D., Bolt E.L. Mechanistic insights into lhr helicase function in DNA repair. Biochem. J. 2020; 477:2935–2947. 32706021 10.1042/BCJ20200379 PMC7437997

[41] Ledger E.V.K. , Lau K., Tate E.W., Edwards A.W. XerC is required for the repair of antibiotic- and immune-mediated DNA damage in *Staphylococcus aureus*. Antimicrob. Agents Chemother. 2023; 67:e0120622. 36802166 10.1128/aac.01206-22 PMC10019262

[42] Sharma P. , Haycocks J.R.J., Middlemiss A.D., Kettles R.A., Sellars L.E., Ricci V., Piddock L.J.V., Grainger D.C. The multiple antibiotic resistance operon of enteric bacteria controls DNA repair and outer membrane integrity. Nat. Commun. 2017; 8:1444. 29133912 10.1038/s41467-017-01405-7 PMC5684230

[43] Clegg S.J. , Jia W., Cole J.A. Role of the *Escherichia coli* nitrate transport protein, NarU, in survival during severe nutrient starvation and slow growth. Microbiology. 2006; 152:2091–2100. 16804183 10.1099/mic.0.28688-0

[44] Spector M.P. , Del Portillo F.G., Bearson S.M.D., Mahmud A., Magut M., Finlay B.B., Dougan G., Foster J.W., Pallen M.J The rpoS-dependent starvation-stress response locus stiA encodes a nitrate reductase (narZYWV) required for carbon-starvation-inducible thermotolerance and acid tolerance in *Salmonella typhimurium*. Microbiology. 1999; 145:3035–3045. 10589711 10.1099/00221287-145-11-3035

[45] Weber M. , Burgos R., Yus E., Yang J.S., Lluch-Senor M., Serrano L. Impact of C-terminal amino acid composition on protein expression in bacteria. Mol. Syst. Biol. 2020; 16:e9208. 32449593 10.15252/msb.20199208 PMC7246954

[46] Rossi S.C. , Barrientos-Moreno L., Paone A., Cutruzzolà F., Paiardini A., Espinosa-Urgel M., Rinaldo S. Nutrient sensing and biofilm modulation: the example of L-arginine in *Pseudomonas*. Int. J. Mol. Sci. 2022; 23:4386. 35457206 10.3390/ijms23084386 PMC9028604

[47] Cunin R. , Eckhardt T., Piette J., Boyen A., Piérard A., Glansdorff N. Molecular basis for modulated regulation of gene expression in the arginine regulon of *Escherichia coli* K-12. Nucleic Acids Res. 1983; 11:5007–5019. 6348703 10.1093/nar/11.15.5007 PMC326233

[48] Reslane I. , Halsey C.R., Stastny A., Cabrera B.J., Ahn J., Shinde D., Galac M.R., Sladek M.F., Razvi F., Lehman M.K. et al. Catabolic ornithine carbamoyltransferase activity facilitates growth of *Staphylococcus aureus* in defined medium lacking glucose and arginine. Mbio. 2022; 13:e0039522. 35475645 10.1128/mbio.00395-22 PMC9239276

[49] Cunin R. , Glansdorff N., Piérard A., Stalon V. Biosynthesis and metabolism of arginine in bacteria. Microbiol. Rev. 1996; 50:314–352. 10.1128/mr.50.3.314-352.1986 PMC373073 3534538

[50] Yeom J.H. , Go H., Shin E., Kim H.-L., Han S.H., Moore C.J., Bae J., Lee K. Inhibitory effects of RraA and RraB on RNAse E-related enzymes imply conserved functions in the regulated enzymatic cleavage of RNA. FEMS Microbiol. Lett. 2008; 285:10–15. 18510556 10.1111/j.1574-6968.2008.01205.x

[51] Bechhofer D.H. , Deutscher M.P. Bacterial ribonucleases and their roles in RNA metabolism. Crit. Rev. Biochem. Mol. Biol. 2019; 54:242–300. 31464530 10.1080/10409238.2019.1651816 PMC6776250

[52] Bonnefoy V. , Pascal M.C., Ratouchniak J., Chippaux M. Autoregulation of the nar operon encoding nitrate reductase in *Escherichia coli*. Mol. Gen. Genet. 1986; 204:180–184. 3091994 10.1007/BF00330207

[53] Boecker S. , Slaviero G., Schramm T., Szymanski W., Steuer R., Link H., Klamt S. Deciphering the physiological response of *Escherichia coli* under high ATP demand. Mol Sys Biol. 2021; 17:e10504. 10.15252/msb.202110504 PMC8686765 34928538

[54] Lee H. , Popodi E., Tang H., Foster P.L. Rate and molecular spectrum of spontaneous mutations in the bacterium *Escherichia coli* as determined by whole-genome sequencing. Proc. Natl. Acad. Sci. U.S.A. 2012; 109:E2774–E2783. 22991466 10.1073/pnas.1210309109 PMC3478608

[55] Fogle C.A. , Nagle J.L., Desai M.M. Clonal interference, multiple mutations and adaptation in large asexual populations. Genetics. 2008; 180:2163–2173. 18832359 10.1534/genetics.108.090019 PMC2600949

[56] Lauxen A.I. , Kobauri P., Wegener M., Hansen M.J., Galenkamp N.S., Maglia G., Szymanski W., Feringa B.L., Kuipers O.P. Mechanism of resistance development in *E. coli* against TCAT, a trimethoprim-based photoswitchable antibiotic. Pharmaceuticals (Basel). 2021; 14:392. 33919397 10.3390/ph14050392 PMC8143356

[57] Card K.J. , Thomas M.D., G. Jr, J.L. B., Lenski R.E Genomic evolution of antibiotic resistance is contingent on genetic background following a long-term experiment with *Escherichia coli*. Proc. Natl. Acad. Sci. U.S.A. 2021; 118:e2016886118. 33441451 10.1073/pnas.2016886118 PMC7865137

[58] Keogh D. , Tay W.H., Ho Y.Y., Dale J.L., Chen S., Umashankar S., Williams R.B.H., Chen S.L., Dunny G.M., Kline K.A. Enterococcal metabolite cues facilitate interspecies niche modulation and polymicrobial infection. Cell Host Microbe. 2016; 20:493–503. 27736645 10.1016/j.chom.2016.09.004 PMC5076562

[59] Liu X. , Li Y., Guo Y., Zheng Z., Li B., Wood T.K., Cai X., Wang X. Physiological function of Rac prophage during biofilm formation and regulation of rac excision in *Escherichia coli* K-12. Sci. Rep. 2015; 5:16074. 26530864 10.1038/srep16074 PMC4632033

[60] Bessaiah H. , Anamale C., Sung J., Dozois C.M. What flips the switch? Signals and stress regulating extraintestinal pathogenic *Escherichia coli* type 1 fimbriae (Pili). Microorganisms. 2021; 10:5. 35056454 10.3390/microorganisms10010005 PMC8777976

[61] Corvec S. , Caroff N., Espaze E., Reynaud A. 11 Mutation in the ampC promoter increasing resistance to beta-lactams in a clinical *Escherichia coli* strain. Antimicrob. Agents Chemother. 2002; 46:3265–3267. 12234856 10.1128/AAC.46.10.3265-3267.2002 PMC128767

[62] Fournier B. , Lu C.Y., Lagrange P.H., Krishnamoorthy R., Philippon A. Point mutation in the pribnow box, the molecular basis of beta-lactamase overproduction in *Klebsiella oxytoca*. Antimicrob. Agents Chemother. 1995; 39:1365–1368. 7574532 10.1128/aac.39.6.1365 PMC162743

[63] Bonnefoy V. , Demoss J.A. Nitrate reductases in *Escherichia coli*. Antonie Van Leeuwenhoek. 1994; 66:47–56. 7747940 10.1007/BF00871632

