# Supplementary material for: Clinically relevant mutations in regulatory regions of metabolic genes facilitate early adaptation to ciprofloxacin in Escherichia coli
Source: Nucleic Acids Res. 2024 Aug 24;52(17):10385–99. doi: 10.1093/nar/gkae719 (PMC11417348; doi:10.1093/nar/gkae719)
Supplement: gkae719_Supplemental_Files [file gkae719_supplemental_files.zip › Supplementary data NAR.pdf]

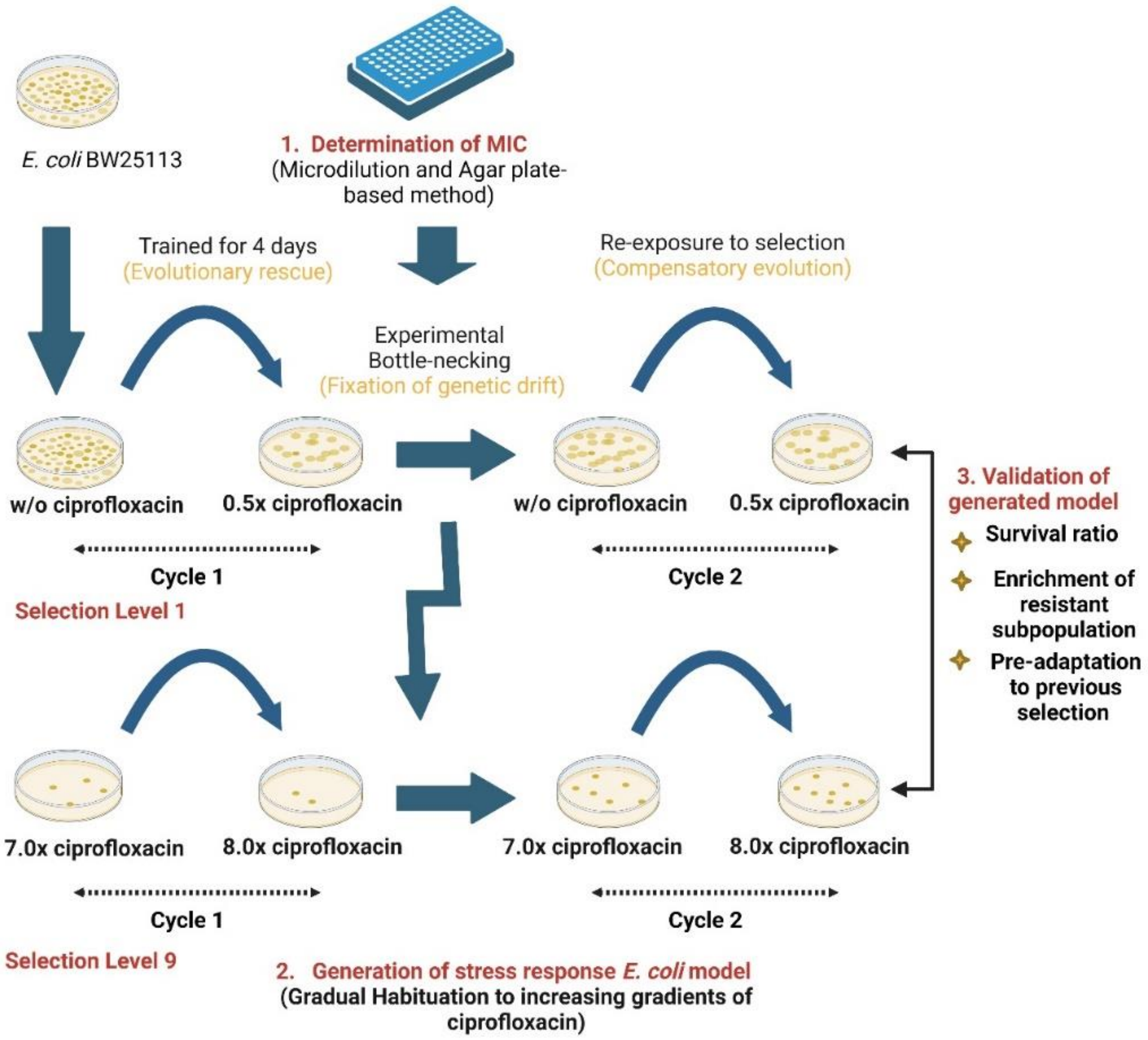

Supplementary Fig.1: Schematic representation of adaptive-stress response model used in this study.

A

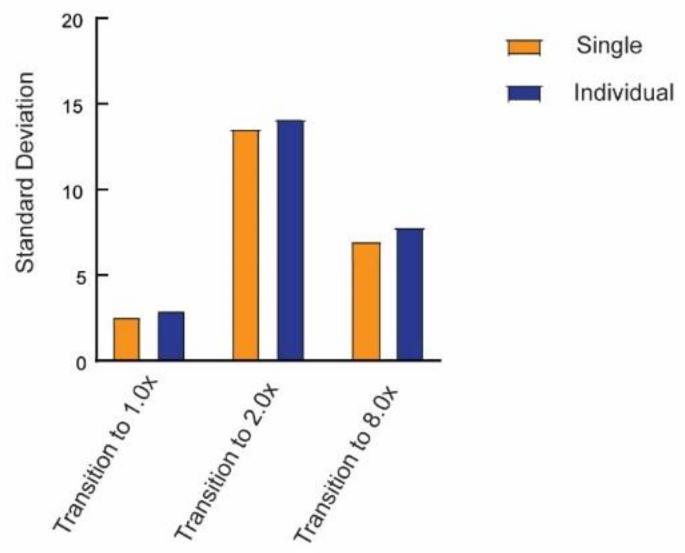

B

Properties	Naive	0.5x survived	1.0x survived	2.0x survived	8.0x survived
Maximum Growth Rate (μmax)	1.26 ± 0.04	1.22 ± 0.03	1.30 ± 0.04	1.22 ± 0.05	** 0.94 ± 0.02
Fitness coefficient (W)	1.00 ± 0.03	0.97 ± 0.02	1.03 ± 0.03	0.97 ± 0.03	** 0.74 ± 0.02

Supplementary Fig.2: A. Luria-Delbruck fluctuation assay: Standard deviation values of colony counts for single and individual cultures plotted against transition to different selection levels. **B. Fitness cost assay:** Maximum growth rate (μmax) and fitness co-efficient (W) estimations for naïve and subpopulations adapted to different selection levels from growth kinetics data calculated using a widely used method described earlier (Durso *et al*. 2004). ** indicates p<0.005 in paired t-test.

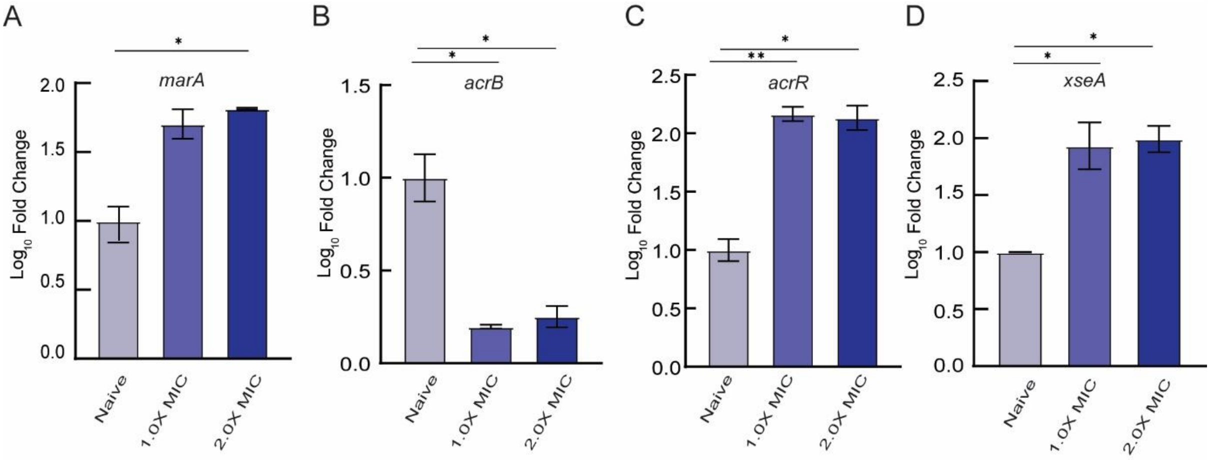

Supplementary Fig.3: A. – D. Transcript levels of *marA*, *acrB*, *acrR*, and *xseA* (expressed Log_{10} fold change) estimated for naïve and subpopulations adapted to 1.0x and 2.0x MIC. * p<0.05, and ** p<0.005 in paired t-test.

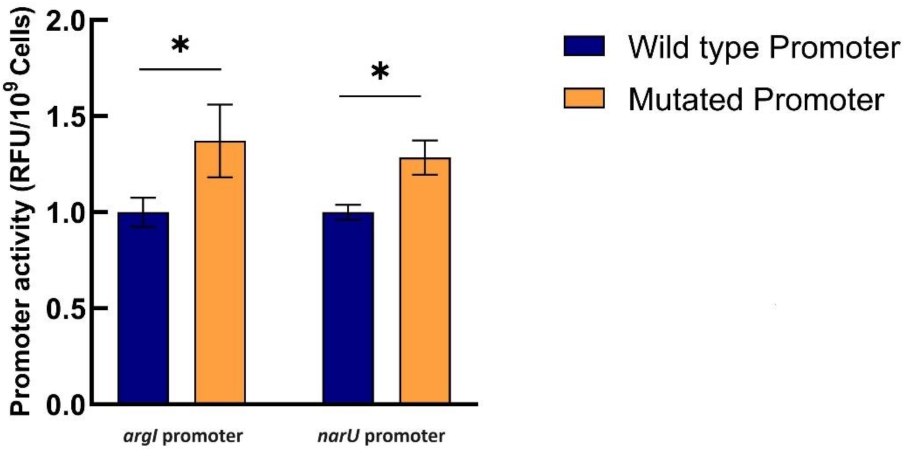

Supplementary Fig. 4: Determination of promoter activity by reporter assay. Wild type and mutated promoter along with ~100 bp flanking regions of both *argI* and *narU* promoters were cloned upstream to GFPmut3.1 reporter gene in the pRU1097 vector. Promoter activity was determined by measuring fluorescence (Ex/Em: 485nm/510nm). Fluorescence in RFU/10^9 cells was plotted along Y-axis. Mutations in the upstream regulatory region of both *argI* (G4468182A) and *narU* (T1538580C) resulted in significantly the respective higher promoter activity compared to that of wild type promoters. * $p<0.05$ in paired t-test.

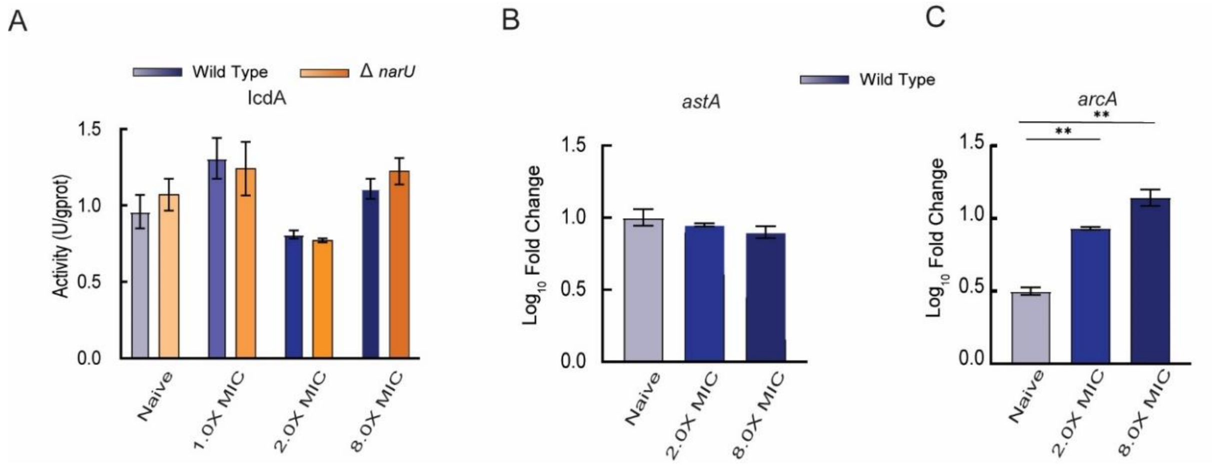

Supplementary Fig.5: Activity of isocitrate dehydrogenase (IcdA), transcript levels of arginine succinyltransferase (*astA*) and arginine deiminase (*arcA*) A. Comparable activities (U/gram of protein) of IcdA in naïve and adapted subpopulations of wild type and Δ*narU E. coli* indicated comparable basal energy flux through Tricarboxylic acid (TCA) cycle. **B.** Comparable transcript levels of *astA* (expressed as Log_{10} fold change) in naïve and supra-MIC ciprofloxacin adapted subpopulations indicate no role for aerobic arginine catabolism in adaptation and **C.** Higher transcript levels of *arcA* (expressed as Log10 fold change) in supra-MIC ciprofloxacin adapted subpopulations with respect to naïve indicate that the ADI (Arginine deiminase) pathway is pivotal for arginine catabolism under ciprofloxacin stress. * p<0.05, **p<0.005 and *** p<0.001 in paired t-test.

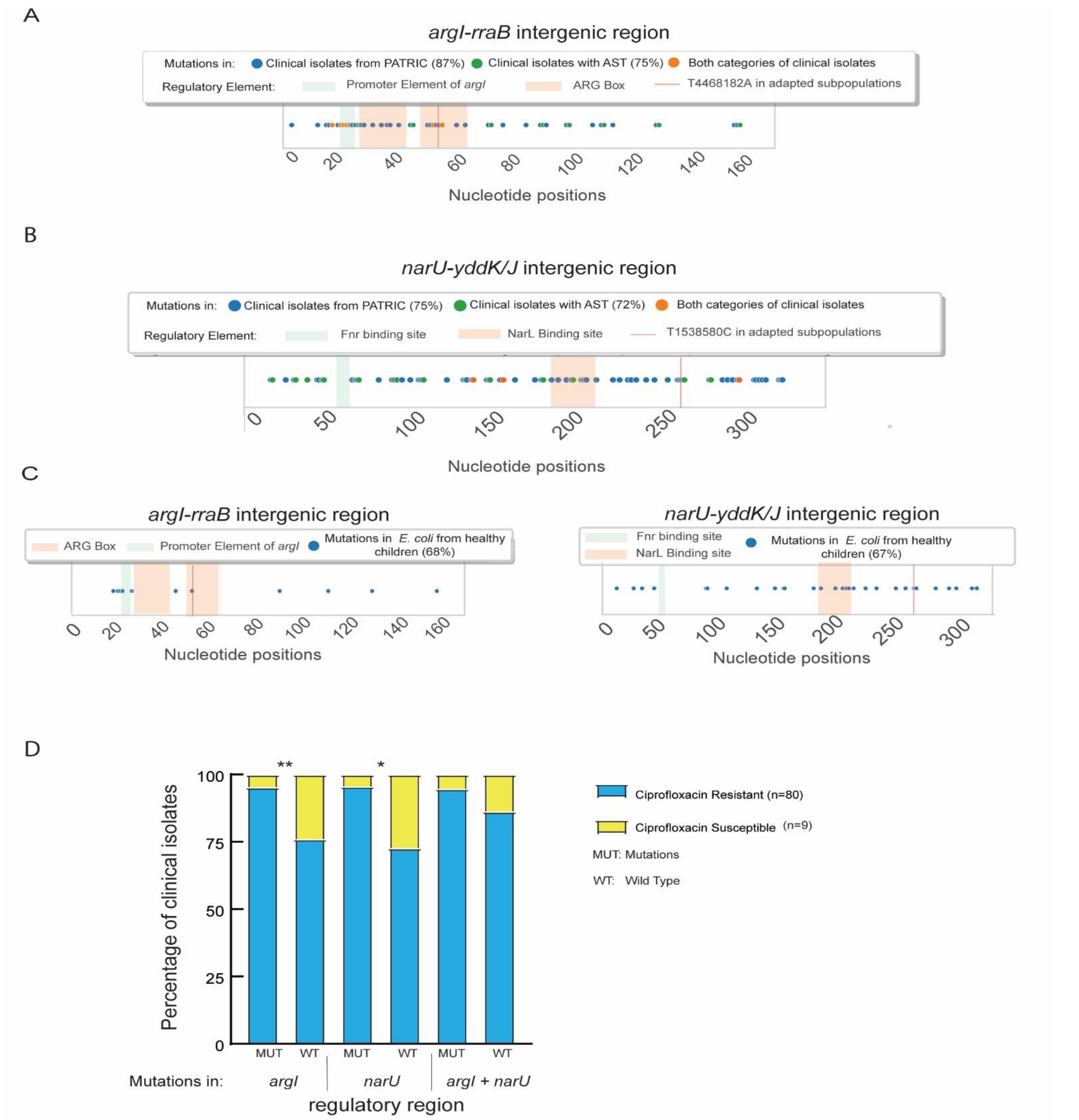

Supplementary Fig.6: Probing of *argI-rraB* and *narU-yddK/J* intergenic regions from clinical isolates for the presence of mutations. A. - C. Map of the *argI-rraB* and *narU-yddK/J* intergenic regions showing the distribution of mutations in clinical isolates of *E. coli* from PATRIC database (n=661; no information on antimicrobial susceptibility is available) (**A.**), those with AST (antimicrobial susceptibility testing) profile (n=89) (**B.**), and fluoroquinolone-resistant *E. coli* from healthy children (n=113) (**C.**). Blue-, green-, and orange-coloured solid circles indicate nucleotide positions of mutations. The percentage of whole-genome sequences harbouring mutations in *argI-rraB* and *narU-yddK/J* intergenic region is indicated in parentheses. **D.** Bar diagrams showing mutations in *argI* regulatory (*argI-rraB* intergenic) and *narU* regulatory (*narU-yddK/J intergenic*) region are significantly enriched among ciprofloxacin-resistant clinical isolates of *E. coli* (n=80) compared those susceptible to ciprofloxacin (n=9). WT of *argI* + *narU* indicated neither of the regulatory region of *argI* and *narU* or only one of them harbours mutations. **p*<0.05, and ***p*<0.005 in Fischer's exact test.

Supplementary Table 1: Primer sets use in this study

**A. Primer sequences used for Sanger sequencing**			
**S.l No.**	**Primer Name**	**Sequence (5'-3')**	**Amplicon size (bp)**
1	*narU* F	TGGGGATTAAGTAGCCAG	218
2	*narU* R	GGAAAACGATCTGTCCAG	
3	*marR* F	GGGTCGCTTAATCCATATGG	471
4	*marR* R	CCTCGATCCAGTCCAAAATG	
5	*lhr* F	AGCGAATTGGCCTTTCTG	298
6	*lhr* R	CCCCGCGAATTAGTAAAGAC	
7	*rraB* F	CTGGCGTGAAATCGAGTA	336
8	*rraB* R	CTGCGGAAAGATGGTGTT	
9	*e14* F	CCGGTCAGGACAAAGTAA	473
10	*e14* R	GTGTTATACGCCCGTTTC	
11	*rac* F	GTTTGCAGGATATCGTCACG	585
12	*rac* R	TCCCCTCTAATCCGTACCAT	
13	*t-arg* F	CTGGTGCCTGCGGTTAATA	480
14	*t-arg* R	TGCTAAGGGAGTATGCGGT	
15	F1	GAAGAAGCTTGATTTAACTTATTG	245
16	F2	ATCACCTCCGCTATATGTAAA	
17	R1	TCAGTTTATTTGTTGGCTTAATGT	398
18	R2	TTTAATTTTCATGCTGCTTTCCTT	

**B. Primer sequences used in qPCR assays**		
**Sl. No.**	**Primer Name**	**Sequence (5'-3')**
1	*qmarA*F	ACTGGAGAAAGTGTCAGAGC
2	*qmarA*R	CAGATAGAGTATCGGCTCGT
3	*qacrB*F	CTGAACTGGCGAAGATGGA
4	*qacrB*R	AAAGGTCCCGAGCAATACC
5	*qacrR*F	AGTGAGATCTGGGAACTGTC
6	*qacrR*R	CTCCATCAATAATCGACGCC
7	*qxseA*F	CTCTGCCGGTGATCATCTA
8	*qxseA*R	GGAATGCGGCTGGTAAAA
9	*qargI*F	CAGCAATGCAATCCGTTC
10	*qargI*R	GGTGGGAATATTACGCTGAC
11	*qadiA*F	GGTCTATATGGTGCCAAGC
12	*qadiA*R	GTCATAGGTGCAGTTGGTC
13	*qastA*F	ACGAACACGGCTATTCAC
14	*qastA*R	GATGTACCTGACCGATGAC
15	*qarcA*F	GCGTTGATGTTCCTGACT
16	*qarcA*R	CAGATTCATGGTACGGGAC
17	*qrraB*F	GTACACCATCGAACACCATC
18	*qrraB*R	GATGTCGCAGCAAATCAC
19	*qnarU*F	CTCACATGCACACATTGG
20	*qnarU*R	CTCTGACGGAATAGATGTGG
21	*qnarZ*F	GGTGCCGTAGTAGTTAAAGC
22	*qnarZ*R	TGGTTCGGAAGTAACTGG
23	*qnarY*F	ACATATTCCTACTGCTCGCC
24	*qnarY*R	CTGCCCGTACATCTCCTTTT
25	*qnarW*F	TACGGATTTCACCCACAACC
26	*qnarW*R	CTTTTCATACTCCGCCAGCA
27	*qnarV*F	CATCTCTGACGATCCACCAC
28	*qnarV*R	CACTACGCCGGATATCATCA
29	*qg6pdF*	TGCCTTCCCTGTATCAAC
30	*qg6pd*R	CTCAGGGTGTCCCATAAA
31	*qaldB*F	CGACCGATACCTGATTGT
32	*qaldB*R	GGTGACCACCTTCAAAAC
33	*qldhA*F	GCTGCCTGAGAATCAATC
34	*qldhA*R	CCAACCCTGTTCTCTGAA
35	*qfimB*F	GCTTTTCAACAACGCACCC
36	*qfimB*R	GAATCTCCAGTGACAACCCG

Supplementary Table 1: Primer sets use in this study

37	*qfimA*F	GTTGCATCTAAAGCCGCTG
38	*qfimA*R	GATCTGCACACCAACGTTTG
39	*qentB*F	GGGGTATATGCCCACATT
40	*qentB*R	GGTGCTGGCAGTAATTCTTC
41	*16srRNA*F	AACACATGCAAGTCGAACG
42	*16srRNA*R	CCTACTAGCTAATCCCATCTGG
**C.**	**Primer sequences used in promoter cloning**	
**Sl. No.**	**Primer Name**	**Sequence (5’-3’)**
1	*argI*_KpnI F	TGCAGGTACCACTTCGAAAGAGCATCG
2	*argI*_SalI R	TGTAGTCGACAACGCTTCAACTGCTGC
3	*narU*_KpnI F	AAGTGGTACCGCAGTGCCATATTGTTC
4	*narU*_SalI R	TCTAGTCGACACCTTCTGGACAGATCG
**D.**	**Primer sequences used in site-directed mutagenesis**	
**Sl. No.**	**Primer Name**	**Sequence (5’-3’)**
1	*argI*_SDMF	TTCAATTTATATGGAT**A**ATTATTCATTTGCAAG
2	*argI*_SDMR	CTTGCAAATGAATAAT**T**ATCCATATAAATTGAA
3	*narU*_SDMF	ATCGTGAATCTAAAGGGT**C**ACATATTAACTATA
4	*narU*_SDMR	TATAGTTAATATGT**G**ACCCTTTAGATTCACGAT
**E.**	**Primer sequences used in knock-out generation**	
**Sl. No.**	**Primer Name**	**Sequence (5’-3’)**
1	tRNA_KO_FP	TCCGCTACCTCTTCGATACCTTCATTGCTGAGATTTCGGAGTGTAGGCTGGAGCTGCTTC
2	tRNA_KO_RP	AGCGGTCGGAGGTTCGAATCCTCCCGGATGCACCATATTCATTCCGGGGATCCGTCGACCTG

N.B. –

For site-directed mutagenesis primers: the substituted nucleotides are written in bold letter

For knock-out primers: the underlined regions indicate priming sites whereas the rest of the primers refer to homology extension sites

Supplementary Table 4: The quality control values related to sequence reads and mapping to Reference Genome *Escherichia coli* BW25113 (Gene Accession Number: CP009273.1)

**Serial No.**	**Barcode No.**	**Experimental Set**	**NanoPlot Data**			**QualiMap Data**	
			**Read Quality**			**Mapping Quality**	
			**Mean**	**Median**	**N50 length (Kb)**	**Mean**	**Coverage (X)**
01	01	Naïve (1)	12.4	12.5	26.144	58.57	121.15 ± 14.96
02	02	Naïve (2)	12.4	12.4	21.588	58.54	109.59 ± 13.83
03	03	0.5x Survived (1)	12.3	12.4	22.338	58.50	86.17 ± 12.95
04	04	0.5x Survived (2)	12.3	12.4	13.123	58.50	192.65 ± 19.56
05	05	1.0x Survived (1)	12.2	12.2	12.814	58.33	89.64 ± 12.52
06	06	1.0x Survived (2)	12.4	12.4	12.276	58.55	232.61 ± 31.89
07	07	2.0x Survived (1)	12.5	12.5	16.926	58.54	221.39 ± 26.90
08	08	2.0x Survived (2)	12.3	12.3	17.441	58.51	128.28 ± 21.85
09	12	8.0x Survived	12.3	12.3	10.230	58.50	168.19 ± 19.22
**Average:**			**12.34**	**12.38**	**16.987**	**58.50**	**149.96 ± 19.30**

Supplementary Table 5: Sanger sequencing data (Mutations and the corresponding wild type sequences in Naive) generated in this study

Serial Number	Gene Accession Number	Gene	Identified in
1	OQ354662	Truncated ATP-dependent helicase (*lhr*)	1.0x MIC
2	OQ354663	Wild type ATP-dependent helicase (*lhr*)	Naive
3	OQ354664	Wild type repressor of multiple antibiotic resistance (*marR*)	Naive
4	OQ354665	Mutated repressor of multiple antibiotic resistance (*marR*)	1.0x MIC
5	OQ354666	Truncated repressor of multiple antibiotic resistance (*marR*)	2.0x MIC
6	OQ354667	isocitrate dehydrogenase C (*icdC*) with inverted repeat	2.0x MIC
7	OQ354668	Wild type sequence of *narU* upstream element	Naive
8	OQ354669	Mutated sequence with a SNP at *narU* upstream element	1.0x MIC onwards
9	OQ354670	Wild type sequence of *narU* upstream element	Naive
10	OQ354671	Mutated sequence with a SNP at *rraB* upstream element	8.0x MIC
11	OR141528	Mutated tRNA-cytidine(32) 2-sulfurtransferase (*ttcA*)	2.0x MIC
12	OR187533	Mutated t-RNA-Arg (anticodon: ACG)	2.0x MIC
13	OR133307	Inverted *fimS*-IRL	2.0x MIC onwards
14	OR133308	Inverted *fimS*-IRR	2.0x MIC onwards
