# Supplementary material for: Clinically relevant mutations in regulatory regions of metabolic genes facilitate early adaptation to ciprofloxacin in Escherichia coli
Source: Nucleic Acids Res. 2024 Aug 24;52(17):10385–99. doi: 10.1093/nar/gkae719 (PMC11417348; doi:10.1093/nar/gkae719)
Supplement: gkae719_Supplemental_Files [file gkae719_supplemental_files.zip › Supplementary table legends.docx]

**Supplementary Table 1:** Primer sets used in this study

**Supplementary Table 2:** Clinical dataset (Whole genome assemblies; n=661) retrieved from PATRIC database

**Supplementary Table 3:** Clinical dataset (Whole genome assemblies) along with AST profile (n=89) analysed in this study (data from CMC, Vellore)

**Supplementary Table 4:** The quality control values related to sequence reads and mapping to Reference Genome *Escherichia coli* BW25113 (Gene Accession Number: CP009273.1)

**Supplementary Table 5:** Sanger sequencing data (Mutations and the corresponding wild type sequences in Naive) generated in this study
